# Site-specific HPV18 integration facilitates cervical carcinogenesis through metabolic reprogramming-induced dysfunction of the SpHK1/S1P/S1PR1 pathway

**DOI:** 10.1038/s41419-025-08195-7

**Published:** 2026-01-09

**Authors:** Liming Wang, Xiaomin Li, Ci Ren, Liting Liu, Jiaying Yao, Min Wu, Hui Shen, Da Zhu, Xiaoli Wang, Zan Yuan, Yafei Huang, Hui Wang

**Affiliations:** 1 Zhejiang Key Laboratory of Precision Diagnosis and Therapy for Major Gynecological Diseases, Women’s Hospital, School of Medicine, Zhejiang University, Hangzhou, Zhejiang China; 2 Department of Anesthesiology, Hubei Key Laboratory of Geriatric Anesthesia and Perioperative Brain Health, and Wuhan Clinical Research Center for Geriatric Anesthesia, Tongji Hospital, Tongji Medical College, Huazhong University of Science and Technology, Wuhan, Hubei China; 3 Department of Obstetrics and Gynecology, Tongji Hospital, Tongji Medical College, Huazhong University of Science and Technology, Wuhan, Hubei PR China; 4 Department of Gynecologic Oncology, Women’s Hospital, School of Medicine, Zhejiang University, Zhejiang, China; 5 Annoroad Gene Technology (Beijing) Co. Ltd, Beijing, China; 6 Department of Pathogen Biology, School of Basic Medicine, Tongji Medical College, Huazhong University of Science and Technology, Wuhan, Hubei China; 7 State Key Laboratory for Diagnosis and Treatment of Severe Zoonotic Infectious Diseases, Huazhong University of Science and Technology, Wuhan, Hubei China; 8 Key Laboratory of Organ Transplantation, Ministry of Education; NHC Key Laboratory of Organ Transplantation; Key Laboratory of Organ Transplantation, Chinese Academy of Medical Sciences; Organ Transplantation Clinical Medical Research Center of Hubei Province, Wuhan, Hubei China

**Keywords:** Cancer metabolism, Cervical cancer

## Abstract

Integration of high-risk human papillomavirus into specific loci of the genome is a pivotal event in cervical carcinogenesis; however, it’s underlying mechanism remains largely undefined. Here, through establishing an *8q24* site-specific HPV18 gene knock-in cell model by utilizing the CRISPR/Cas9 system, we discover that HPV18 knock-in (HPV-KI) results in a global alteration of the genome’s topologically associating domain structure and an up-regulation of cancer-related genes in HPV^-^ HaCaT cells, among which the significantly up-regulated IL-17 signaling pathway and S100A8/A9 are partitularly prominent. Further mechanistic study demonstrate that HPV-KI reprograms metabolic pathway, especially up-regulates glycolysis and subsequently facilitates glycerolipid synthesis in HaCaT cell, leading to sphingosine-1-phospate (S1P) secretion and enhanced SpHK1/S1P/S1PR1 signaling pathway, thereby activating the the MAPK and NF-κB signaling pathways followed by inducing the expression of S100A8/A9, and hence induces the malignant transformation of cells. Importantly, inhibition of the S1P/S1PR1 signaling pathway down-regulates the expression of S100A8/A9 and suppresses the growth of HPV-KI cells and xenograft derived from cervical cancer patient. These findings provide novel insights into HPV integration-induced cervical carcinogenesis and identify potential therapeutic targets for its treatment.

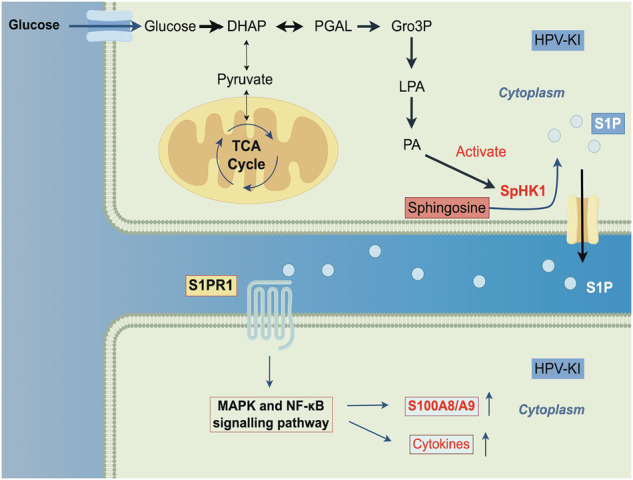

## Introduction

Most women of childbearing age commonly experience genital HPV infection, which is often transient. However, a small percentage of individuals with persistent infections may progress to cervical intraepithelial neoplasia (CIN) or even cervical cancer, with high-risk HPV (HR-HPV) integration playing a crucial role in this process. Indeed, HR-HPV integration results in aberrant expression of host genes and genomic instability [1, 2], a hallmark of cancer [3]. Further investigations have confirmed *8q24* as a “hotspot” of HPV integration in cervical cancer that can cause an interaction between HPV and the oncogene *c-MYC* [4, 5]. In addition to oncogene induction, other host biological alterations such as metabolic reprogramming and chronic inflammation that play a crucial role in the development of cervical cancer may also be induced by the integration of HR-HPV; however, the precise underlying mechanisms remain incompletely illustrated.

During the development of cancer, there is an up-regulation in aerobic glycolysis, which supplies rapid energy for tumor cell metabolism. Moreover, the intermediate metabolites generated in this process also participate in de novo lipogenesis and lipd remodeling [6], which not only provide glycerophospholipids for membrane homeostasis, but also improve bioactive lipid synthesis followed by evoking intracellular signaling by binding to G-protein-coupled receptors, thereby resulting in carcinogenesis [7, 8]. Previous studies have confirmed the regulatory role of oncogenic E6 and E7 in activating metabolic pathways and directly influencing enzymes involved in the glycolysis pathway, thereby promoting the warburg effect through increased glucose uptake, activation of glycolysis, and pentose phosphate pathway (PPP) [9]. Furthermore, the bioactive lipid pathways such as arachidonic acid-derived COX-2/PEG-2, which are facilitated by the activation of glycolysis, have been shown to link inflammation with carcinogenesis during cervical cancer development [10]. However, the extent to which HR-HPV integration affects oncogenic metabolic reprogramming during cervical carcinogenesis remains to be elucidated. Therefore, additional investigations are requied to fill this knowledge gap. Accordingly, identifying the pathways involved in this process could potentially help to discover new therapeutics or preventive targets for cervical cancer.

In this study, we developed a site-specific HPV18 knock-in HaCaT (HPV-KI/HaCaT) cell model to explore the mechanisms underlying HPV integration and host cell transformation in cervical carcinogenesis. By utilizing various approaches, we observed an increase in glycolysis and glycerolipid remodeling in HPV-KI/HaCaT cells. Furthermore, the SpHK1/S1P/S1PR1 pathway, downsteam of glycerolipid remodeling, was found to play a significant role in the malignant transformation induced by HPV-KI. Our findings suggest that SpHK1/S1P/S1PR1 may serve as a promising target for the treatment of cervical cancer.

## Results

### Site-specific HPV18 knock-in induces malignant transformation in HPV^-^ HaCaT cells

**Fig. 1:**
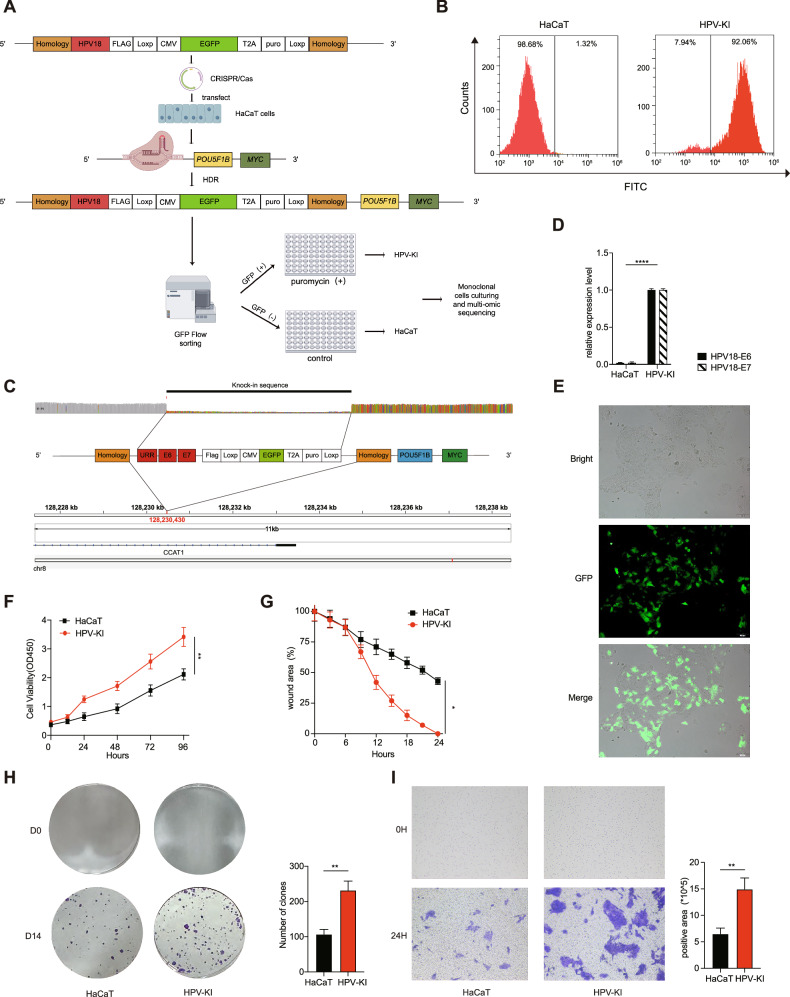
Establishment of a site-specific HPV18 gene knock-in at the *8q24* locus cell model and characterization of the altered biological properties of HPV-KI. **A** Schematic representation of the *8q24* site-specific HPV18 gene knock-in strategy using the CRISPR-Cas9 system. **B** Representative flow cytometric plot showing the proportions of EGFP-positive cells in HPV-KI and HaCaT cells. **C** Detection of site-specific HPV18 knock-in through whole-genome long read sequencing. **D** The relative mRNA expression levels of HPV18 E6 and HPV18 E7 in HaCaT and HPV-KI. The primers for qPCR detection are listed in Supplementary Table 1. **E** Representative immunofluorescence images showing EGFP expression in HPV-KI cells. **F** The viability of different groups of cells at 0 h, 12 h, 24 h, 48 h,72 h and 96 h, as detected by CCK-8 assay. **G** The relative wound healing rates of HaCaT and HPV-KI cells were measured. **H** Representative images (left) and quantitative analysis results (right) showing the colony-forming ability of HaCaT and HPV-KI cells in day 14. **I** Representative images (left) and quantitative analysis results (right) showing the invasion ability of HaCaT and HPV-KI cells, determined using transwell assays at 24 h after culture. The data shown represents mean ± SD (Student’s *t*-test). *P* value is denoted as *P < 0.05, **P < 0.01, and ***P < 0.001, ****P < 0.0001, “ns” represents “not significant”. **A** and **C** were created with Figdraw.com.

To explore the potential effect of HPV integration on the development of cervical cancer, we established stable *8q24* site-specific HPV18 gene knock-in cell lines using HaCaT as the parent through employing CRISPR/Cas9 gene editing combined with homologous end repair [11]. The knock-in cell lines harbored the Flag-Loxp+CMV-EGFP-2A-Puro-polyA+Loxp cassette and HPV18 URR-E7-E6 genes, which were flanked by 1000 bp homologous arm sequences (Fig. 1A). HaCaT represented the negative control cells for HPV-KI (HPV knock-in). Subsequently, screening with puromycin and single cell fluorescence-activated cell sorting (FACS) were performed to obtain EGFP-positive monoclonal cells (Fig. 1B). The inserted sequence was validated by whole-genome long read sequencing (Fig. 1C), which revealed a 3.7 kb insertion at chr8: 128230431, characterizing the donor DNA insertion at position *8q24*. Furthermore, the inserted sequence matched the donor DNA sequence, indicating HPV18 knock-in into the target locus of HaCaT. Of note, the discrepancy between the total number of reads (n = 46) and the number of partially matched provider DNA reads (n = 9) indicate that the allele is present as a heterozygote in the inserted cell lines (9/46). Additionally, E6 and E7 mRNA expression along with EGFP were detected in HPV-KI cell lines (Fig. 1D, E and Supplementary Table 1). These results together demonstrate the successful construction of HaCaT cell with HPV18 gene knocked in at *8q24* locus.

Next, we asked whether HPV knock-in alters the biological properties of HaCaT cells. As shown in Fig. 1F, HPV-KI exhibited faster growth compared to the control HaCaT, as measured using a CCK-8 assay. We also observed an increased clone formation capacity of HPV-KI compared to HaCaT (Fig. 1H). In addition, transwell and wound healing assays revealed that the invasive and migrating capacity of HPV-KI cells were significantly enhanced in comparison to that of HaCaT (Fig. 1G, I and Supplementary Fig. 1A). Thus, through establishing HPV18 site-specific knock-in cell lines, we reveal that HPV knock-in changes the biological properties of HaCaT cells, driving them toward malignant transformation.

### The TAD score is significantly changed and cancer-related genes are enriched in HPV-KI cells

**Fig. 2:**
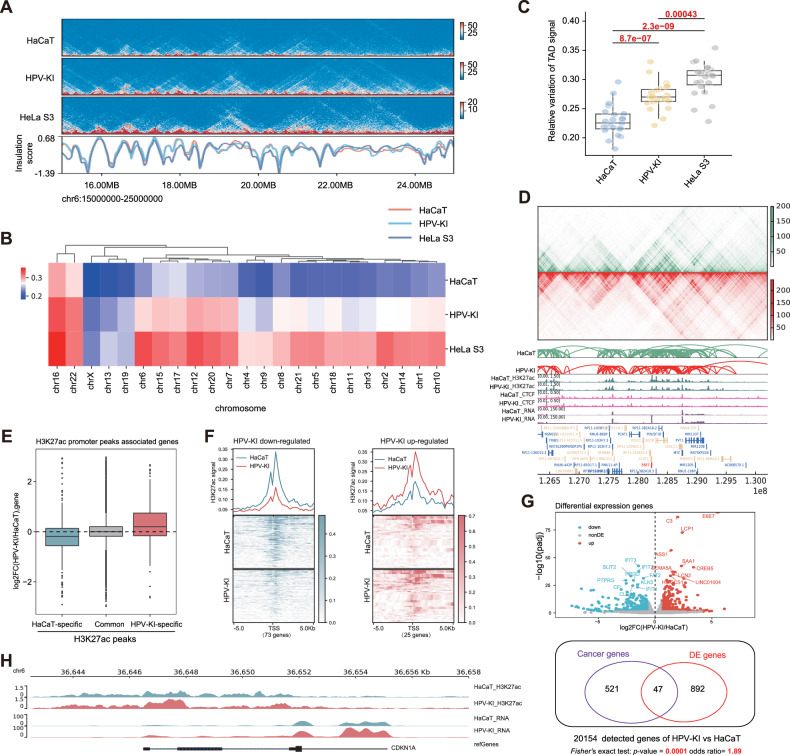
HPV-KI exhibits a higher propensity for malignancy compared to HaCaT at the spatial genomic and transcriptional levels. **A** Snapshot of the interaction heat map for high-order chromatin structures in HaCaT, HPV-KI and HeLaS3 (Chr6:15.00–25.00 Mb). Heatmap (**B**) and boxplot (**C**) of TAD signal distribution among HaCaT, HPV-KI and HeLaS3, where each dot represents a chromosome. The Wilcoxon test was used to assess significant differences in TAD signal variation between groups. **D** Hi-C contact heatmap at HPV18 knock-in loci and neighboring regions (Chr8:126.3–130.2 Mb, resolution: 20 kb) as well as matching tracks of the H3K27ac and CTCF ChIP-seq profile in HPV-KI/HaCaT. The gene track is shown in the bottom panel. **E** A boxplot shows log_2_ Foldchange of gene expression between HPV-KI and HaCaT, focusing on HaCaT-specific H3K27ac peaks’ target genes, common H3K27ac peaks’ target genes and HPV-KI-specific H3K27ac peaks’ target genes. **F** H3K27ac signal profiles demonstrating the down-regulated and up-regulated genes which are positively correlated with H3K27ac alterations signal depicted in **E**. **G** The upper panel is a volcanic plot of differential expression genes, and the lower panel is a *Fisher’*s exact test between DE genes and cancer-related genes (HPV-KI vs HaCaT). **H** BrowserTrack plots at *CDKN1A* gene (chr6: 36642500-36658000), revealing higher chromatin H3K27ac signals and transcriptional RNA levels in HPV-KI.

Next, Hi-C sequencing was performed in both HPV-KI and HaCaT cells to interrogate the genomic changes resulted from HPV-KI. Of note, although the transactivation domain and topology associated domain (TAD) are generally considered to be relatively stable across different cell types, some TAD reorganizations have been observed during cellular differentiation. We thus utilized TAD signal variance to investigate TAD reprogramming following HPV knock-in. The relative variance of TAD signal was higher in HPV-KI compared to HaCaT, recapitulating those observed in HeLa S3, a cell line that also has HPV18 integration at *8q24* (Fig. 2A–C and Supplementary Table 2) [12].

We further investigated the reprogramming of 3D chromatin structure at the knock-in locus and its vicinity. No significant alteration was found in TAD boundary and loop within the genomic region of HPV insertion and adjacent areas in HPV-KI and the control. CTCF plays a crucial role as a primary organizer of 3D chromatin architecture, responsible for establishing chromatin loops and structuring TAD, while H3K27ac is a histone modification for active enhancer [13]. Thus, we analyzed H3K27ac and CTCF ChIP profiles in HPV-KI/HaCaT cell lines, and identifed changes in CTCF looping and transcriptional activation resulted from HPV knock-in. However, no significant changes were observed in H3K27ac loops or CTCF binding sites at the HPV knock-in locus or nearby regions, which is consistent with the results of transcriptomic analysis (Fig. 2D).

H3K27ac histone modification is regarded as a active marker associated with transcription. Therefore, we asked if it is true in the context of HPV-KI-induced alteration. To test this, we first identified differential H3K27ac peaks between HPV-KI and HaCaT by using MAnorm software [14], and then divided H3K27ac peaks that are located in promoter regions(±2 kb) around transcription start site (TSS) into HPV-KI-specific peaks, common peaks and HaCaT-specific peaks. As expected, the log_2_ Foldchange of gene expression between HPV-KI and HaCaT was positively correlated with H3K27ac specific peaks (Fig. 2E). Specifically, 25 up-regulated genes with increased promoter H3K27ac peak signals and 73 down-regulated genes with decreased promoter H3K27ac peak signals were detected in HPV-KI cells (Fig. 2F).

Next, differentially expressed genes (DEGs) were analyzed using a cutoff of |log_2_ Foldchange | ≥1 and *q* < 0.05, resulting in the identification of 939 DEGs in HPV-KI compared to HaCaT. Moreover, we employed a cancer-related gene set (https://cancer.sanger.ac.uk/census) to intersect the DEGs, and found that cancer-related genes were significantly enriched in DEGs between HPV-KI and HaCaT (Fig. 2G and Supplementary Table 3). Moreover, we compared these DEGs to those between CIN or early stage CESC and normal epithelial cells identified by single-cell sequencing [15], and found that 40 and 42 DE genes are shared between HPV-KI/HaCaT and CIN/normal, and HPV-KI/HaCaT and early stage CESC/nomal, respectively (Supplementary Fig. 2A), indicating that the HPV knock-in cell model can reflect the characteristics of cervical cancer development in vivo. This notion is further supported by the finding that *CDKN1A*, a gene closely related to cervical carcinogenesis, showed significantly increased H3K27ac peaks and increased transcription in HPV-KI cells (Fig. 2H), suggesting their higher propensity for malignancy at the transcriptional level.

### HPV knock-in up-regulates multiple oncogenic pathways that are enriched in human cervical cancer and K14-HPV mouse

**Fig. 3:**
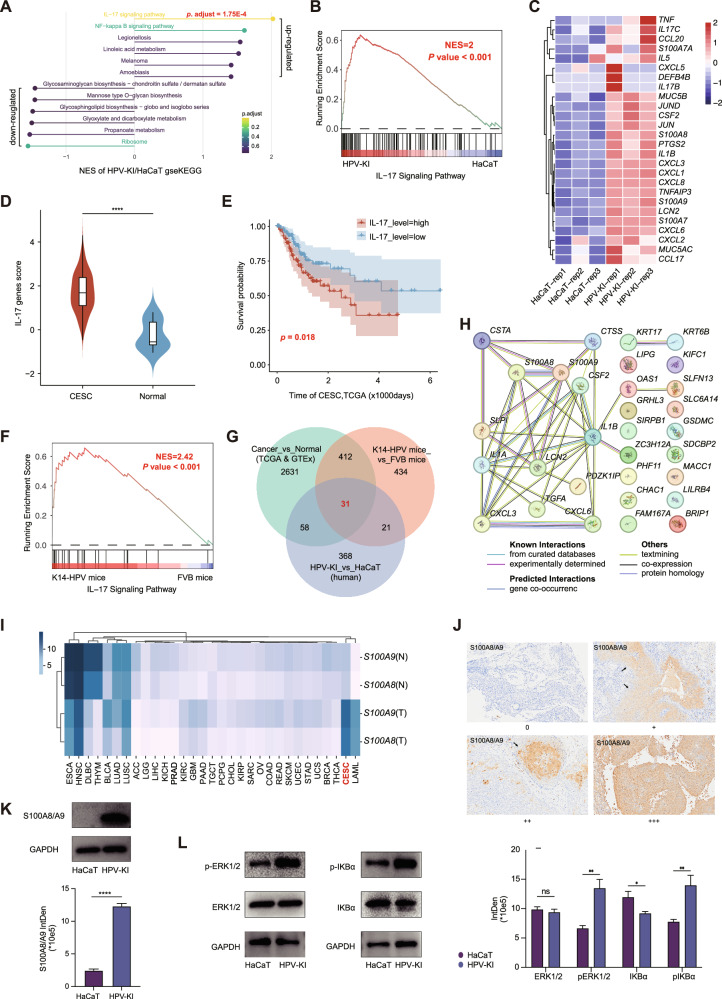
The IL-17 signaling pathway and S100A8/A9 are up-regulated in cervical cancer and HPV-related models. **A** KEGG enrichment for genes up- and down-regulated in HPV-KI/HaCaT cells. **B** GSEA analysis for IL-17 signaling pathway in HPV-KI and HaCaT cells. **C** Heatmap displaying up- and down-regulated genes of the IL-17 signaling pathway in HPV-KI and HaCaT cells. **D** Comparison of IL-17 signaling pathway gene scores between CSCE and normal control in TCGA and GTEx database. **E** Kaplan–Meier survival analysis of CESC patients with high and low IL-17 gene scores. **F** GSEA analysis of the IL-17 signaling pathway in RNA-seq data from swollen ears of K14-HPV mice compared to normal FVB controls. Venn diagram illustrating the number (**G**) and STRING network visualization of 31 up-regulated genes (**H**) among HPV-KI/HaCaT cells, K14-HPV mice/FVB, and CSEC/normal control. **I** The TCGA database was utilized to assess the expression levels of *S100A8* and *S100A9* in different cancers and their corresponding normal tissues, with “*S100A8*(T)”, “*S100A8*(N)”, “*S100A9*(T)”, and “*S100A9*(N)” representing the respective expression levels in tumor and normal tissues. **J** Immunohistochemistry demonstrating different expression levels of S100A8/A9 in CC samples. **K** S100A8/A9 protein levels in HPV-KI/HaCaT cells. **L** Western blot analysis of ERK1/2, IκBα, phosphorylated ERK1/2 and phosphorylated IκBα protein levels in HPV-KI/HaCaT cells, with GAPDH serving as control. The data shown represent mean ± SD (Student’s *t*-test). *P* value is denoted as *P < 0.05, **P < 0.01, and ***P < 0.001, ****P < 0.0001, “ns” represents “not significant”.

To identifiy pathways that are involved in HPV-KI-induced malignant transformation, we performed KEGG functional annotation and gene set enrichment analysis (GSEA) on HPV-KI and HaCaT cells, finding that the IL-17 signaling pathway is the most up-regulated pathway in HPV-KI cells, followed by the NF-κB pathway (Fig. 3A–C, Supplementary Table 4). Of note, in HPV-KI cells, *IL17RA* and *IL17A* were underexpressed (Cycle Threshold>30), and *IL17RC* expression did not change significantly (Supplementary Tables 1, 5). Nevertheless, analysis of the TCGA and GTEx databases comfirmed that the expression of the IL-17 signaling pathway is elevated in cervical squamous cell carcinoma (CESC) compared to normal cervical tissue (*p* = 7.5E-07) (Fig. 3D and Supplementary Fig. 3D). Furthermore, survival analysis demonstrated that high expression of IL-17 signaling pathway-related genes was associated with a poor prognosis for cervical cancer patients (Fig. 3E).

The K14-HPV16 mice represent a transgenic mouse model in which the early region of HPV16 is knock-in into the genome of FVB mice and under the control of the human *Keratin14* promoter. These transgenic mice exhibit epidermal hyperplastic lesions that progress to dysplastic lesions [16]. RNA-sequencing was conducted on the swollen ear tissue from K14-HPV16 transgenic mice, with FVB mice as control group. The analysis also revealed a significant up-regulation of the IL-17 signaling pathway (Fig. 3F and Supplementary Table 6). Together, the up-regulation of IL-17 signaling pathway induced by HPV knock-in can represent the charactristics shared by cervical cancer patients with poor prognosis and K14-HPV16 mice.

In a parallel approach, we performed another KEGG enrichment analysis on the 939 DEGs between HPV-KI and HaCaT cells. Again, the up-regulated DEGs were significantly enriched in IL-17 pathway, along with MAPK, NF-κB, and TNF pathways (Supplementary Fig. 3A–C). The integrated analysis also revealed a significant enrichment of DEGs in NF-κB, TNF, cancer, IL-17 and MAPK pathways (Supplementary Fig. 3A). Similarly, prediction of transcription factors (TF) identified RELA, NFKB1, JUN, and SP1 as key regulatory TFs involved in the regulation of these DEGs (Supplementary Fig. 3B) [17]. Therefore, these findings together indicate the potential involvement of these pathways in the HPV-KI-induced malignant transformation. Furthermore, these pathways may potentially interact with each other in this process, as indicated by the shared “hub genes” among them in the protein-protein interaction network (PPI) analysis (Supplementary Fig. 3C). More interestingly, analysis of RNA-seq data from TCGA, RNA-seq of K14-HPV16 transgenic mice and HPV-KI/HaCaT revealed 31 synchronously up-regulated genes, including *S100A8/A9*, *CXCL3/6*, *IL1B* and *LCN2*, which are all “hub genes” shared by the four pathways (Fig. 3G, H).

### Overexpression of S100A8/A9 is associated with the transformation of HPV-KI and promotes cervical tumorigenesis

Among these hub genes, *S100A8* and *S100A9* immediately gained our attention given their reported involvement in the regulation of the immune microenvironment and affecting patient response to immunotherapy in CESC [18]. Indeed, *S100A8/A9* were identified as DEGs during cervical tumorigenesis in epithelial cells [15]. Our pan-cancer analysis of *S100A8/A9* expression using TCGA and GTEx data also revealed the significantly higher expression of *S100A8/A9* in tumor regions than in adjacent normal tissues in cervical cancer. Notably, *S100A8/A9* demonstrated more specific expression in cervical cancer tumor regions compared to other cancer types (Fig. 3I and Supplementary Fig. 3E). Previous scRNA data revealed predominant expression of *S100A8/A9* in epithelial cells and monocytic myeloid-derived suppressive cells (M-MDSCs) [15]. We confirmed this expression pattern by immunohistochemical (IHC) analysis of specimens from cervical cancer patients, showing the predominant S100A8/A9 expression n epithelial cells and myeloid cells. Importantly, although changes in S100A8/A9 protein levels across different FIGO stages were not significant, cases with vascular involvement and lymphatic metastasis showed higher levels of S100A8/A9 expression (Fig. 3J, Supplementary Tables 7, 8). Therefore, these findings collectively indicate the role of S100A8/A9 in the carcinogensis of cervical cancer.

In consistent with these findings and the up-regulated transcriptional expression of S100A8/A9 in HPV-KI compared to HaCaT cells (Fig. 3G, H), S100A8/A9 expression at protein level was also higher in HPV-KI cells (Fig. 3K). It’s important to note that as an inflammatory mediator, S100A8/A9 also interacts with various tumor signaling pathways such as toll-like receptor (TLR) and receptor for advanced glycation end products (RAGE), and can form a positive feedback loop with MAPK, NF-κB and other signaling pathways, thereby promoting the proliferation, migration and invasion of tumor cells [19]. Therefore, we went on to examine the activation of the MAPK and NF-κB signaling pathways in HaCaT/HPV-KI cells, and found significantly increased phosphorylated ERK1/2 and IκBα proteins in HPV-KI cells (Fig. 3L), indicating that HPV knock-in activates MAPK and NF-κB signaling pathways in addition to increasing the expression of S100A8/A9. Moreover, key TFs of DEGs including S100A8/A9 are associated with these two signaling pathways (Supplementary Fig. 3B). Thus, these results together demonstrate that S100A8/A9 may form a positive feedback loop as previously reported in HPV-KI-induced malignant transformation, and offer an explanation for the discrepancy between up-regulated IL-17 pathway and unchanged or even down-regulated expression of *IL17RC*, *IL17RA*, and *IL17A* in HPV-KI cells (Fig. 3A-C, Supplementary Tables 1, 5).

### Glycolysis and lipid metabolic reprogramming in HPV-KI and cervical cancer

**Fig. 4:**
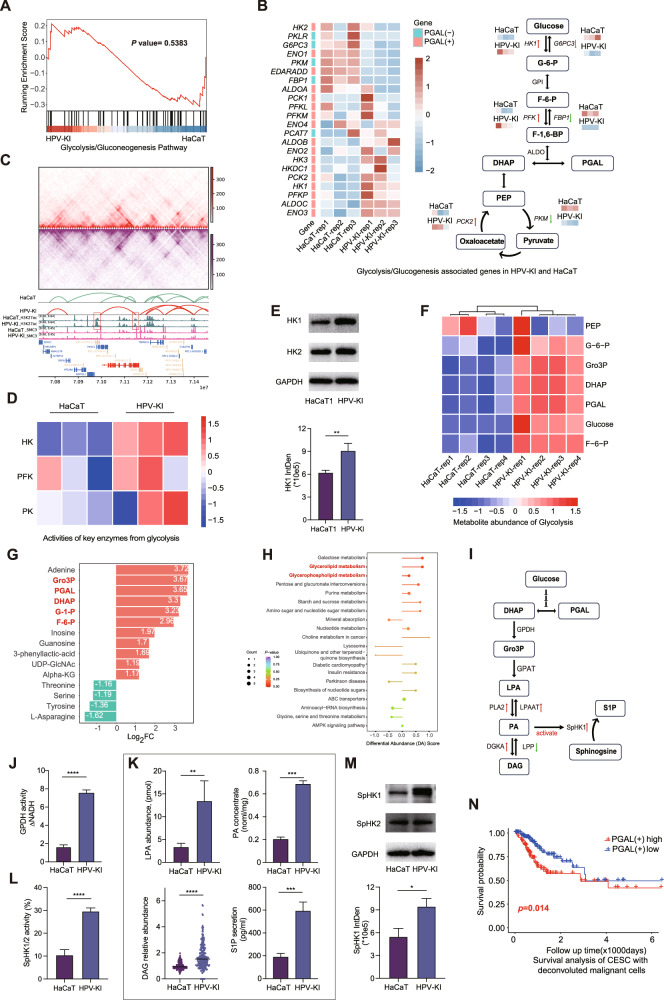
Glycolysis and lipid metabolic reprogramming in HPV-KI compared to HaCaT cells. **A** GSEA Analysis based on the gene expression of Glycolysis/Gluconeogenesis Pathway in HPV-KI/HaCaT cells. **B** Gene expression and heatmap of Glycolysis/Glucogenesis associated genes were analyzed in HPV-KI and HaCaT. The green box in the heatmap indicates genes favoring PGAL consumption, while the pink box indicates genes involved in PGAL accumulation. **C** Hi-C contact heatmap at *HK1* neighboring regions (chr10:71078602-71161637) was performed along with matching tracks of the ChIP-seq profile. The gene track is shown in the bottom panel. Red box indicates the H3K27ac peak of TSS and TES of *HK1*. **D** Heatmap analysis revealing the activity levels of HK, PFK and PK in HPV-KI/HaCaT cells. **E** Western blot depicting HK1/2 expression levels in HPV-KI/HaCaT cells, with GAPDH serving as control. **F** Metabolite abundance involved in Glycolysis/Glucogenesis between HPV-KI and HaCaT is visualized using a heatmap. **G** Boxplot illustrating the top fold changed metabolites associated with carbon metabolism in HPV-KI/HaCaT. Metabolites associated with glycolysis are highlighted in red bold. **H** KEGG analysis of metabolomic changes between HPV-KI and HaCaT base on DA score of carbon metabolism. **I** Diagram illustrating how Gro3P connects glycolysis to de novo GL synthesis, while SpHK1 is regulated by PA and links GL synthesis to sphingosine metabolism. **J** Assessment of GPDH activity in HPV-KI/HaCaT cells. **K** Quantification of LPA abundance, PA concentration, relative abundance of DAG in HPV-KI/HaCaT cells. Additionally, the S1P concentration in the culture medium supernatant is presented. **L** Assessment of SpHK1/2 activity in HaCaT and HPV-KI is shown. **M** Western blot showing SpHK1/2 expression levels in HPV-KI/HaCaT cells, with GAPDH serving as control. The data are calculated by two-tailed Student’s *t*-test. *P* value is denoted as *P < 0.05, **P < 0.01, and ***P < 0.001, ****P < 0.0001, “ns” represents “not significant”. **N** The overall survival of CESC cases from the TCGA cohort is stratified into high PGAL(+) and low PGAL(+) score groups using Kaplan-Meier analysis.

We next sought to explore the pathways that are upstream of or in parallel to the upregulation of NF-κB/MAPK-S100A8/A9 axis in HPV-KI-induced malignant transformation. The malignant transformation of cells is often accompanied by an augmented demand for rapid energy and materials for biosynthesis, resulting in elevated aerobic glycolysis [20]. Indeed, glyco-metabolic pathway exhibited a tendency to be up-regulated in HPV-KI compared to HaCaT cells in KEGG pathway analysis (Fig. 3A, Fig. 4A and Supplementary Table 4), and further correlation analysis found that genes involved in glycolysis such as *HK1*, *PFK* and *PCK2* were significantly up-regulated, while those participate in promoting gluconeogenesis including *G6PC3*, *FBP1* and *PKM* were down-regulated (Fig. 4B). Consistently, Hi-C and H3K27ac/CTCF ChIP analyses profiles indicated a higher level of H3K27ac peak signals in TSS and Transcription End Site (TES) of *HK1* gene in HPV-KI compared to HaCaT, while no changes in H3K27ac peak signals at *G6PC3* locus were detected (Fig. 4C and Supplementary Fig. 4A). Next, we exmamined the activity of HK, PFK and PK, which are key enzymes in glycolysis, revealing a significant increase in HK activity but not PFK and PK in HPV-KI cells (Fig. 4D and Supplementary Fig. 4B). Additionally, we observed up-regulation of the isoenzyme HK1 but no difference in HK2 protein expression in HPV-KI/HaCaT (Fig. 4E). Thus, these results together indicates that HPV knock-in facilitates glycolysis while inhibits gluconeogenesis (Fig. 4B).

Dysregulated glucose metabolism is often accompanied by the perturbation of other metabolisms. To systemically profile metabolic reprogramming in HPV-KI, a carbon metabolism quantification assay was conducted. As expected, we observed marked changes in glucose metabolism in HPV-KI, with metabolites involved in glycolysis such as glucose, G-6-P, F-6-P, DHAP, PGAL and glycerol-3-phosphate (Gro3P) showing significant increases, while amino acids, including L-asparagine, threonine and tryrosine decreased (Fig. 4F, G, Supplementary Fig. 4C and Supplementary Table 11). Next, KEGG enrichment analysis based on Differential Abundance (DA) Score was employed to assess the overall changes in all metabolites. The most significantly enriched pathways were glycerophospholipid (GPL), glycerolipid (GL) and galactose metabolism (Fig. 4H and Supplementary Table 13), suggesting the changes in lipid metobolism. Indeed, we found a significant reprogramming of lipid metabolism in HPV-KI compared to HaCaT cells, as examined by quantitative lipidomics analysis. In HPV-KI cells, 601 lipid products increased, while 54 products exhibited a decrease compared to HaCaT. The increasing lipid products covered most lipid subclasses (Supplementary Fig. 4D, E and Supplementary Table 12), with the most notable increase observed in TGs (Supplementary Fig. 4F). KEGG analysis based on DA score of lipid metabolites revealed enrichment in pathways such as thermogenesis, vitamin digestion/absorption and cholesterol metabolism (Supplementary Fig. 4G and Supplementary Table 14).

Having found the dysregulation of both glucose and lipid metabolisms in HPV-KI cells, we next explored the possible underlying mechanism. Dihydroxyacetone phosphate (DHAP) and glyceraldehyde triphosphate (PGAL) are the two intermediate metabolites in glycolysis that play crucial roles in bridging glycolysis with other metabolic pathways. Specific for lipid metabolism, glycerol-3-phosphate dehydrogenase (GPDH, isoform: GPD1/GPD1L) catalyzes the conversion of DHAP into Gro3P, which is subsequently converted into lysophosphatidic acid (LPA) by glycerol-3-phosphate acyltransferase (GPATs) followed by transforming into phosphatidic acid (PA). This process represents the initial step in the de novo synthesis of glycerophospholipids (GPL) and triacylglycerols (TG). (Fig. 4I) [21]. Therefore, we next measure GPDH activity, which indicated the coversion of DHAP into Gro3P, in HPV-KI and HaCaT cells, and revealed an increased catalytic activity in HPV-KI (Fig. 4J). Moreover, quantified PA assay and Lipid-omics analysis demonstrated increased levels of LPA, PA and diglyceride (DAG) in HPV-KI cells (Fig. 4K). Therefore, these results, together with the profound up-regulation of lipid metabolism (Supplementary Table 12), indicate the increased lipid biogenesis following glycolysis induced by HPV konock-in.

A previous study has reported that PA acts as an activator of sphingosine kinase 1 (SphK1), retaining it to the membrane and activating its catalytic activity to promote the production of sphingosine-1-phosphate (S1P) from sphingosine [22, 23] through the phosphorylation of sphingosine, a reaction catalyzed by SphK1 at the plasma and membrane, and by SphK2 at the endoplasmic reticulum (ER), mitochondria, and nucleus. Upon stimulation, S1P generated at the cytoplasm and membrane is released via specific transporters and binds to specific S1P receptors (S1PRs) to initiate downstream signaling pathways including MAPK and NF-κB pathways (“inside-out” signaling) [24, 25]. Since the latter two pathways and S100A8/9 are all up-regulated in HPV-KI cells, we thus asked if SphK1/2 are also increased. Indeed, increased enzyme activity of SphK1/2 and up-regulation of SphK1 protein levels were both observed in HPV-KI cells (Fig. 4L, M), likely accounting for the heightened secretion of S1P and the subsequent up-regulation of NF-κB/MAPK-S100A8/A9 axis.

Next, we asked if the metabolic reprogramming observed in HPV-KI cells could be similarly found in human cervical cancer. For this, we first performed correlation analysis of the GEPIA2 (http://gepia2.cancer-pku.cn/-correlation) database to investigate the association between the expression of *S100A8/A9* and key enzyme of glucose metabolism in CESC. The expression of *S100A8/A9* showed a positive correlation with *HK1* (R = 0.37, *P* = −2.8E-11) that facilitates glycolysis, and a negative correlation with *G6PC3* (R = −0.47, *P* = −2.4E-18) that promotes gluconeogenesis (Supplementary Fig. 5A and Supplementary Table 9). These results are in agreement with those observed in HPV-KI cells. Next, we categorized enzymes involved in glycolysis/gluconeogenesis and the tricarboxylic acid cycle (TCA) cycle into two sets based on their functions in promoting PGAL production (PGAL (+)) or facilitating PGAL consumption (PGAL (-)) (Fig. 4B), followed by utilizing PGAL(+) scores to assess CESC in the TCGA database and correlating the scores with patient survival. Patients with low PGAL (+) scores exhibited improved overall survival compared to those with high PGAL (+) scores (Fig. 4N), suggesting the involvelment of other metabolic perturbations in addition to changes in glucose metabolism in the development of cervical cancer as well. Futhermore, in CESC, *SPHK1* expression was elevated in tumors compared to normal controls (Supplementary Fig. 5B). Therefore, HPV-KI-induced metabolic reprogramming can mimic that happens in cervical cancer.

### Elevated glycolysis is crucial for HPV-KI-induced malignant transformation, S100A8/A9 up-regulation and lipid reprogramming

**Fig. 5:**
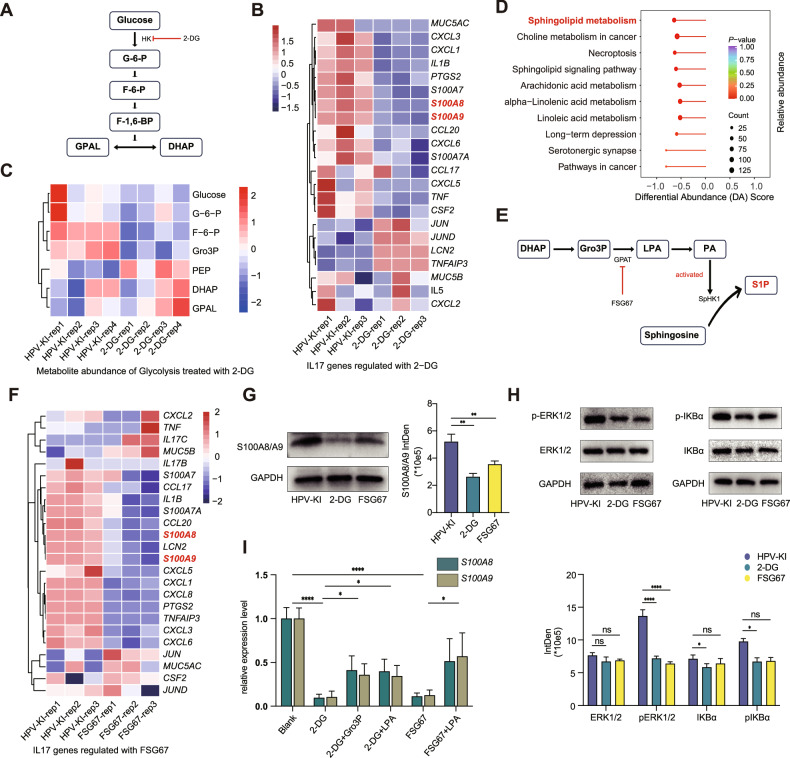
Metabolic reprogramming in HPV-KI is crucial for S100A8/A9 dysregulation. **A** Diagram illustrating 2-DG inhibits HK activity and interferes glycolysis. **B** Heatmap showing the up- and down-regulated genes of IL-17 signaling pathway in 2-DG-treated HPV-KI/HPV-KI. **C** Heatmap of the abundance of metabolites involved in glycolysis between HPV-KI and 2-DG-treated HPV-KI. **D** KEGG analysis reveals Lipid-omics changes between HPV-KI and 2-DG-treated HPV-KI based on the DA Score of the pathway, highlighting annotations for sphingolipid metabolism pathway in red bold. **E** The diagram depicts the de novo synthesis of GL facilitated by DHAP, wherein SpHK1 is activated by PA to enhance S1P secretion. The process is disrupted by inhibition of GPAT by FSG67. **F** Heatmap illustrates up- and down-regulated genes involved in the IL-17 signaling pathway in FSG67- treated HPV-KI/HPV-KI cells. **G** S100A8/A9 protein expression in 2-DG-treated, FSG67-treated and untreated HPV-KI cells. **H** Western blot analysis of ERK1/2, IκBα, phosphorylated ERK1/2, and phosphorylated IκBα protein levels in untreated, 2-DG-treated and FSG67-treated HPV-KI cells. The data are calculated by two-tailed Student’s *t*-test. *P* value is denoted as *P < 0.05, **P < 0.01, ***P < 0.001, and ****P < 0.0001, “ns” represents “not significant”. **I** Expression of S100A8/A9 in 2-DG-treated and FSG67-treated HPV-KI cells replenished with Gro3P and LPA.

Next, we went on to determine the causal relationship of metabolic reprogramming with the malignant transformation and S100A8/A9 up-regulation. Given the crucial role of HK in glucose metabolism and the heightened HK activity in HPV-KI cells, we first selected 2-deoxyglucose (2-DG) to inhibit HK activity, and examined its impact on HPV-KI cells (Fig. 5A). As shown in Supplementary Fig. 6A–C, treatment of HPV-KI with 2-DG markedly deminished cell viability, clone formation and migration of HPV-KI. Moreover, the expression of *S100A8/A9* was notably down-regulated following 2-DG treatment (*S100A8*: Log_2_ Foldchange: −3.51, *P* = 1.17E-13; *S100A9*: Log_2_ Foldchange: −2.93, *P* = 5.23E-43) (Fig. 5B). In line with this, GSEA enrichment analysis revealed a significant down-regulation of the IL-17 signaling pathway in 2-DG-treated HPV-KI cells (Supplementary Fig. 6D and Supplementary Table 17). Finally, western blotting assay comfirmed the significantly reduced S100A8/A9, and phosphorylated ERK1/2 and IκBα levels in 2-DG-treated HPV-KI cells. Collectively, inhibiting glycolysis prevents HPV-KI cells from malignant transformation and up-regulating MAPK/NK-κB- S100A8/9 axis (Fig. 5G, H).

As expected, inhibition of HK led to a decrease in F-6-P and Gro3P abundance; however, DHAP and PGAL as intermediate products did not show significant changes. Moreover, 2-DG reduced the abundance of metabolites involved in TCA cycle, including fumarate, succinate and α-KG, as well as lactate in glycolysis (Fig. 5C, Supplementary Figs. 6F, 7A and Supplementary Table 11). KEGG enrichment analysis based on DA score of carbon metabolism indicated that inhibition of glycolysis promoted PPP and gluconeogenesis in 2-DG-treated HPV-KI cells (Supplementary Fig. 6G and Supplementary Table 15). Importantly, in 2-DG-treated HPV-KI cells, there was a significant alteration in overall lipid metabolism, with most metabolites showing decreased levels (Down:625, up:35) (Supplementary Fig. 6H and Supplementary Table 12). Among all lipid subclasses, TG exhibited the most significant decrease (Supplementary Fig. 7B, C). KEGG enrichment analysis based on DA scores of lipidomics indicated that the sphingolipid metabolism pathway was the most down-regulated pathway (*p* = 2.62E-05), followed by choline metabolism in cancer (*p* = 8.65E-05) (Fig. 5D and Supplementary Table 16). Together, these results demonstate that inhibiting glycolysis diminishes lipid metabolism in HPV-KI cells in addition to preventing these cells from malignant transformation and up-regulating MAPK/NK-κB-S100A8/9 axis.

### Lipid reprogramming acts downstream of glycolysis in HPV-KI-induced malignant transformation and S100A8/A9 up-regulation

Next, we sought to determine if intevening lipid metabolism could recue HPV-KI-induced alterations. GPAT catalyzes the conversion of Gro3P to LPA, followed by catalyzing the acylation of LPA to PA meidiated by lysophosphatidic acid acyltransferase (LPAAT) (Fig. 5E) [26]. There are four GPAT enzymes: GPAT1/2 are localized in the mitochondria, while GPAT3/4 are found in the microsomes. Therefore, we then chose a pan GPAT inhibitor FSG67 to inhibit the conversion of Gro3P/LPA and the de novo synthesis of GPL and TG [21, 27]. As expected, FSG67 treatment induced profound metabolic reprogramming in HPV-KI cells. Although there was no significant change in the abundance of three metabolic intermediates (DHAP, PGAL and Gro3P) in FSG67-treated HPV-KI/HPV-KI, we observed increased levels of 2-phospho-D-glycerate, 3-phosphoglycerate and lactate, with lactate showing the most significant increase (Log_2_ Foldchange: 6.01, *P* value = 0.017). Conversely, metabolites of TCA such as fumarate, succinate and α-KG decreased (Supplementary Fig. 7A, Supplementary Fig. 8A and Supplementary Table 11), while dAMP, dTMP, dCMP, ADP and NAD were significantly increased in both FSG67-treated group. In addition, the levels of xylulose-5-phosphate and D-Ribulose-5-phosphate, two intermediates of pentose phosphate pathway (PPP), were found to be elevated in FSG67-treated HPV-KI (Supplementary Fig. 8A and Supplementary Table 11). We next focused on lipid metabolism. Consistant with previous research, following a 24-hour incubation with FSG67, there was a significant decrease in PA concentration in in FSG67-treated HPV-KI cells [21] (Supplementary Fig. 8B). The abundance of fatty acids (FAs) was profoundly influenced by FSG67, as indicated by a significant reduction in almost all free fatty acids (FFAs) and carnitines (CARs) (Supplementary Fig. 8C).

Finally, we examined the effect of FSG67 treatment on HPV-KI-induced malignant transformation and S100A8/A9 upregulation. The result showed that FSG67 treatment changed the migration but not clone formation properties and cell proliferation of HPV-KI (Supplementary Fig. 6A–C). We also found decreased expression of *S100A8/A9* (*S100A8*: Log_2_ Foldchange, −1.63, *P* = 1.27E-7; *S100A9*: Log_2_ Foldchange, −1.22, *P* = 3.96E-6) (Fig. 5F) and down-regulated IL-17 signaling pathway in FSG67-treated HPV-KI cells (Fig. 5F, Supplementary Fig. 6E and Supplementary Table 18). Similar to 2-DG treatment, FSG67 treatment also reduced the protein levels of S100A8/A9 and phosphorylated ERK1/2 in HPV-KI cells, albeit change of IκBα level was not noted (Fig. 5G, H). Importantly, in 2-DG- and FSG67-treated HPV-KIs, replenishment of Gro3P and LPA both partly reversed the down-regulation of *S100A8/A9* (Fig. 5I, Supplementary Table 1). Taken together, these results indicate that lipid reprogramming acts downstream of glucose metabolism in HPV-KI-induced malignant transformation and up-regulation of MAPK/NK-κB-S100A8/9 axis.

### Targeting S1P/S1PRs down-regulates the expression of S100A8/A9 and inhibits malignant transformation in HPV-KI cells

**Fig. 6:**
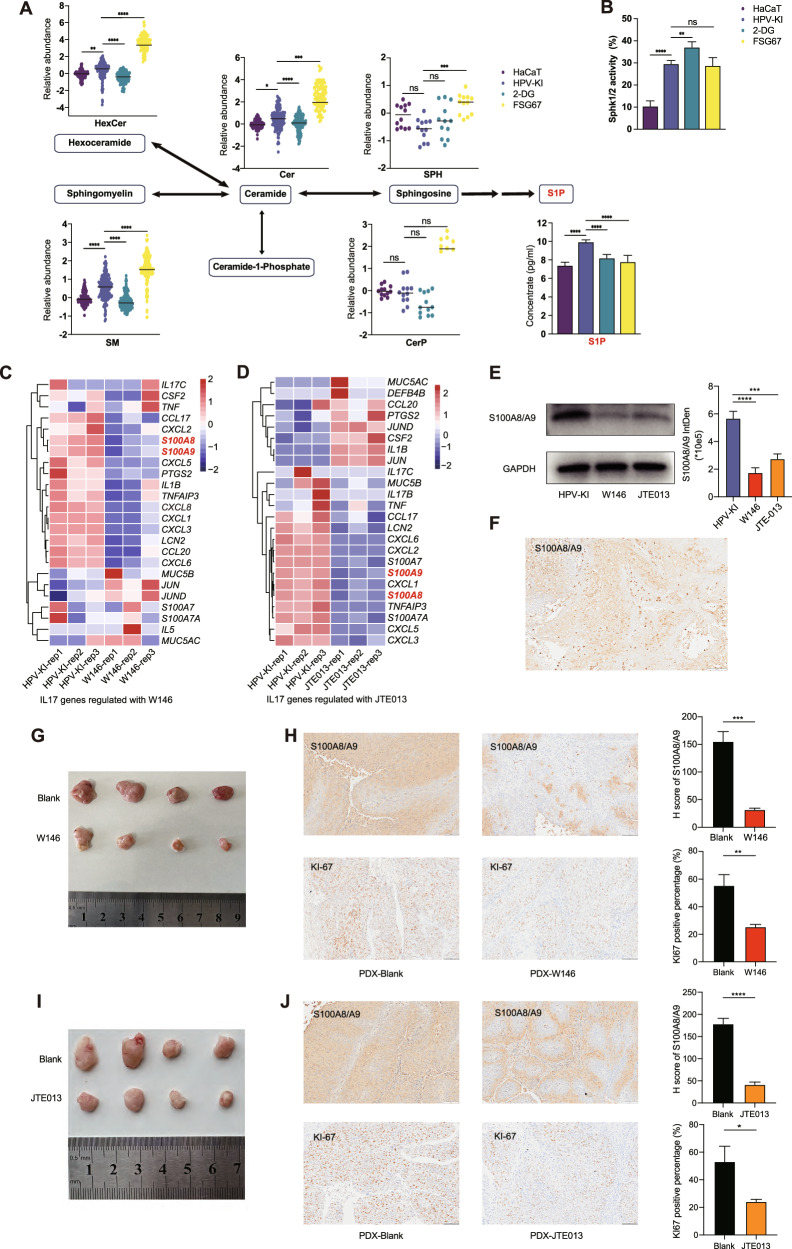
Effects of targeting S1PRs in vitro and vivo. **A** Alterations in D18-sphingolipid metabolites in HaCaT, HPV-KI, 2-DG-treated HPV-KI and FSG67-treated HPV-KI cells. The average abundances of every sphingolipid in HaCaT are normalized to 1, and the fold changes of metabolites in HPV-KI, 2-DG- treated HPV-KI, and FSG67- treated HPV-KI are calculated. The Y-axis represents the log_2_ FC of each D18 sphingolipid metabolite. Statistical significance is determined using a one-way ANOVA-test (*P < 0.05, **P < 0.01, and ***P < 0.001, ****P < 0.0001, “ns” represents “not significant”). **B** Assessment of SpHK1/2 activity in HaCaT, HPV-KI, 2-DG-treated HPV-KI and FSG67-treated HPV-KI is shown. **C** Heatmap illustrating the differential expression of genes involved in the IL-17 pathway between W146-treated HPV-KI and control HPV-KI. **D** Heatmap depicting the differential expression of genes involved in the IL-17 pathway between JTE013-treated and untreated HPV-KI cells. **E** S100A8/A9 protein expression in HPV-KI, W146-treated HPV-KI and JTE013-treated HPV-KI cells. **F** IHC analysis of S100A8/A9 expression in primary tumors from CAT079. **G** Representative image showing PDX tumors from the PBS-treated group and the W146-treated group. **H** The histological characteristics of PBS- and W146-treated PDX tumors. H-score of S100A8/A9 and percentage of Ki-67 positive cells in W146-treated PDX/control are calculated. **I** Image of PDX tumors from DMSO/TWEEN-20/PEG and JTE013- treated group. **J** Evaluation of histological features in PDX tumors following treatment with PBS or JTE013. H-score of S100A8/A9 and percentage of Ki-67 positive cells in JTE013- treated PDX/control are shown.

As previously stated, sphingolipid metabolism pathway, including the production of S1P catalyzed by SphK1/2 and the subsequent S1P/S1PR signaling, is pivotal for the activation of MAPK/NK-κB-S100A8/9 axis. Therefore, we next examined this pathway in HPV-KI cells with or without treatment. S1P secretion assay demonstrated a significant decrease in supernatant of HPV-KI cells after 2-DG treatment. We further investigated changes in the precursors of S1P, such as D18-sphingosine, D18-ceramide, D18-sphingomyelin, and their related metabolites in sphingolipid metabolism. As expected, most D18-sphingolipids increased in HPV-KI compared to HaCaT but decreased significantly in 2-DG-treated HPV-KI cells, while the levels of D18-sphingosines in HPV-KI were lower than those in HaCaT and did not change significantly after 2-DG treatment (Fig. 6A). Additionally, we observed a reduction in PA quantity but unchanged SphK1/2 activity in 2-DG-treated HPV-KI (Fig. 6B, Supplementary Fig. 8B). As for FSG67 treatment, S1P secretion was inhibited in FSG67-treated HPV-KI cells (Fig. 6A). Conversely, the majority of D18-sphingolipids, including hexa-ceramide, sphingomyelin, ceramide and sphingosine showed a significant increase (Fig. 6A). Decreased FFAs and increased sphingolipids accounted for 5 out of 10 and 6 out of 10 top altered metabolites, respectively (Supplementary Fig. 8D). Therefore, inhibition of glucose and lipid metabolism both down-regulate sphingolipid metabolism pathway including the SphK1/2-S1P axis.

Given that intervening glucose and lipid metabolism could have potential side effects, and the observed increase in S1P secretion in HPV-KI cells but a decrease following treatment with 2-DG or FSG67, we opted to target S1PR receptors to assess the role of SphK1/S1P/S1PR pathway in HPV-KI-induced malignant transformation [28]. There are five subtypes of S1PRs, with S1PR1 and S1PR2 being extensively researched. Hence, we chose W146 (a specific inhibitor of S1PR1) and JTE013 (a specific inhibitor of S1PR2), for further investigation.

The CCK-8 assay demonstrated that both W146 and JTE013 significantly suppressed the proliferation of HPV-KI cells (Supplementary Fig. 9A, B). The clone formation and transwell assays also revealed a depressed biological property of W146- and JTE013-treated HPV-KI cells (Supplementary Fig. 9C, D). Subsequently, we investigated the regulation of S100A8/A9 expression by W146 and JTE013. Following treatment with W146 for 24 h, RNA-seq analysis revealed a decrease in the expression of *S100A8/A9* in HPV-KI cells. (*S100A8*: Log_2_ Foldchange, −1.83, *P* = 2.70E-9; *S100A9*: Log_2_ Foldchange, −1.45, *P* = 4.70E-5) (Fig. 6C). In line with this, the KEGG enrichment analysis revealed a down-regulation of the IL-17 signaling pathway (Fig. 6C, Supplementary Fig. 9E and Supplementary Table 19). Similarly, treatment of HPV-KI cells with JTE013 also resulted in down-regulation of *S100A8/A9* expression (*S100A8*: Log_2_ Foldchange, −2.76, *P* = 1.57E-95; *S100A9*: Log_2_ Foldchange, −1.47, *P* = 4.70E-5) (Fig. 6D). GSEA analysis of the IL-17 signaling pathway revealed a decrease, albeit did not reach statistical significance. (Fig. 6D, Supplementary Fig. 9F and Supplementary Table 20). To further ensure that the effect of targeting S1PR can recapitulate that of FSG67 treatment, the DEGs in FSG67-treated HPV-KI were used for intersection with DEGs in W146- and JTE013-treated HPV-KI, resulting in the identification of 204 and 327 shared down-regulated genes, and 70 and 116 shared up-regulated genes, respectively, suggesting the shared downstream signaling pathway of de novo phospholipid synthesis and S1P/S1PR1 receptor pathway (Supplementary Fig. 9G). In addition, KEGG enrichment analysis indicated that these same DEGs were mainly enriched in cytokine-cytokine receptor interaction pathway and IL-17 pathways (Supplementary Fig. 9H).

To further support this notion, at protein level, both W146 and JTE013 reduced S100A8/A9 and phosphorylated ERK1/2 expression in HPV-KI cells, albeit down-regulated expression of phosphorylated IκBα was only noted in W146 but not JTE013 groups (Fig. 6E, Supplementary Fig. 9I). Therefore, targeting S1P/S1PRs can be utilized for counteracting HPV-KI-induced malignant transformation.

### Targeting S1P/S1PRs is effective in treating cervical cancer

Given the crucial role of S100A8/A9 in the development of cervical cancer (Fig. 3I–J, Supplementary Fig. 3E), and the observeved effect of targeting S1P/S1PRs in down-regulating S100A8/A9 expression and reversing HPV-KI-induced malignant transformation, we next asked whether targeting S1P/S1PRs could modulate the tumor development in cervical cancer, as found in HPV-KI cells. For this purpose, we first treated HeLa cells, which also have HPV integration, with W146, followed by comparing the GSEA enrichment of MP6 gene set in W146-treated HeLa cells [18] to that in W146-treated HPV-KI cells. Indeed, the expression of MP6 gene was significantly decreased to the same extent in W146-treated HPV-KI and HeLa cells (Supplementary Fig. 9J).

Next, we utilized a patient-derived xenograft (PDX) model to assess the in vivo efficay of W146 and JTE013 treatment in cervical cancer. The PDX model of patient CAT079 (stage IIB, squamous, G3) (Fig. 6F) was successfully established in NOD/SCID mice. To evaluate the responsiveness of W146, CAT079 PDX mice were treated with either phosphate-buffered saline (PBS) or W146. After 18 days of treatment, tumors were harvested and analyzed. The tumor volumes in the W146-treated group were significantly smaller compared to the PBS group (83.2 mm^3^ *vs* 268.6 mm^3^, *P* = 0.035) (Supplementary Fig. 10A, Fig. 6G). Moreover, IHC analysis demonstrated reduced staining intensity of S100A8/A9 in the W146-treated PDX tumor. Additionally, KI-67 staining demonstrated a decreased percentage of positive cells in the PDX tumors treated with W146 (Fig. 6H). In the JTE013 group, the volumes of PDX tumors in the JTE013-treated group were significantly reduced compared to the control group. (108.5 mm^3^ *vs* 266.8 mm^3^, *P* = 0.017) (Supplementary Fig. 10C, Fig. 6I). The tumors treated with JTE013 exhibited decreased S100A8/A9 staining intensity and a lower proportion of KI-67 positive cells compared to the control group (Fig. 6J). Additionally. the body weight of NOD-SCID mice treated with JTE013 showed a significant reduction (Supplementary Fig. 10D), while W146 had no impact on the body weight of NOD-SCID mice (Supplementary Fig. 10B), suggesting that while both treatments are effective, considering both therapeutic efficacy and safty, W146 may be superior to JTE013 in treating cervical cancer.

## Discussion

HR-HPV integration in human genome has been demonstrated to be related to cervical carcinogenesis. In addition. previous studies have confirmed that there were HPV integration sites with high frequency in human genome in cervical cancer [4]. Howerver, to date, it remains unclear whether all integration events lead to increased levels of virus oncogenes and/or a cell growth advantage, to which host factors also contribute [2]. In our study, we developed an HPV18 site-specific knock-in cell model based on HaCaT, enabling a more in-depth investigation into the mechanisms of malignant transformation during HPV integration.

Overall, the introduction of HPV knock-in results in a more aggressive phenotype in HPV-KI. However, the HPV-*MYC* interaction was not detected in HPV-KI. Heterozygote of HPV-KI was a possible reason for non-detectable HPV-*MYC* interaction. Also, the number of passages may not be sufficient to cause the interaction between HPV and *MYC*. Nevertheless, the alteration of TAD signal variance and increased expression of cancer-related genes in HPV-KI unanimously suggest the malignant transformation of HPV-KI. Therefore, these results together demonstrate that HPV knock-in results in genomic alterations as well as malignant transformation in HPV-KI cells.

The up-regulation of genes in the IL-17 signaling pathway was observed in the HPV-KI/HaCaT system, with *S100A8/A9* being one of the most differentially expressed “hub genes” shared by IL-17, NF-κB and MAPK signaling pathways. These findings are consistent with prior studies that have demonstrated the up-regulation of the *S100A8/A9* and IL-17 signaling pathways during cervical carcinogenesis [15, 29]. Forming a common heterodimer structure, S100A8 and S100A9 have been widely reported to participate in multiple biological processes in tumor cells. As an important inflammatory regulator, the S100A8/A9 complex activates NF-κB and MAPK signaling pathways, playing a crucial role in promoting inflammation during tumorigenesis [30, 31]. Moreover, previous study have reported that NF-κB and MAPK signaling pathways act upstream of S100A8/A9 and are subsequently modulated by S100A8/A9, thereby forming a posive feedback loop [19]. Our study confirmed that the NF-κB and MAPK signaling pathways were activated simultaneously‌ with the up-regulation of S100A8/A9 in HPV-KI cells, suggesting the presence of this feedback loop in the context of HPV integration.

Our HPV-KI model confirms that HPV18 integration induces the dysregulation of glucose metabolism and promotes the metabolites of glycolysis to enter the pathway of de novo synthesis of phospholipids, leading to an extensive metabolic reprogramming of transformed cells. Specifically, HPV-KI exhibited an enrichment of glycolytic metabolites and a greater reliance on GPL and GL for energy support, while HaCaT showed relatively higher levels of metabolites involved in glutamine metabolism and related amino acids, which exhibits a potential dependence on glutamine metabolism. These differential metablic profiles in HPV-KI and HaCaT cells were similar to the those between tumor and normal cells in triple negative breast cancer [32]. In addition, the majority of lipid metabolites showed a notable increase in HPV-KI compared to HaCaT, aligning with the results reported by A. Mukherjee *et al*., suggesting that increased glycolysis provides Gro3P for de novo GPL and GL synthesis in HPV-KI [33]. Therefore, the HPV18 integration-induced metabolic reprogramming also mimics those during cancer development.

In lipid biogenesis, the balance between ceramide and S1P in sphingomyelin metabolism, known as the “sphingolipid rheostat”, plays a crucial role in determining cell fate, including cell proliferation and apoptosis [34]. Specifically, PA activates SpHK1 to facilitate the synthesis of S1P [22], leading to its transportation into the extracellular space and acts on S1PRs in an autocrine or paracrine manner to activate downstream signaling pathways including NF-κB and MAPK signaling pathways [24]. In this study, HPV-KI activates the SpHK1/S1P/S1PRs pathway and the NF-κB/MAPK-S100A8/A9 positive feedback loop. Conversely, S1PRs blockade reverses the up-regulated NF-κB/MAPK-S100A8/A9 axis and malignant transformation in HPV-KI cells. These findings together demonstrate that lipid reprogramming, especially the activated SpHK1/S1P/S1PRs pathway, is responsible for the up-regulation of NF-κB/MAPK-S100A8/A9 axis and malignant transformation induced by HPV integration.

Previous studies have established that S100A8/A9 regulates cell metabolism in the tumor microenvironment, such as carbon metabolism, lipid metabolism, and ROS generation [19]. However, the regulation of S100A8/A9 by oncogenic metabolism has not been thoroughly investigated. Our results instead underscore the regulatory role of glycolysis and de novo GL synthesis in modulating *S100A8*/*A9* and NF-κB/MAPK pathways. In support of this notion, inhibition of glycolysis and lipid biogenesis respectively with 2-DG and FSG67 both markedly down-regulate S1P secretion, S100A8/A9-NF-κB/MAPK axis, and malignant transformation in HPV-KI cells. Therefore, this study reveals a previously unrecognized mechanism by which HPV integration induces elevated glycolysis followed by enhanced lipid biogenesis, leading to the activation of SpHK1/S1P/S1PRs pathway and the subsequent NF-κB/MAPK-S100A8/A9 positive feedback loop, thereby promoting malignant transformation.

It’s important to note that although inhibition of glycolysis and lipid biogenesis have similar effect in the context of HPV integration as demonstrated above, they also result in differential metabolic reprogramming. For example, 2-DG treatment reduces almost all classes of lipid products in HPV-KI cells. However, FSG67-induced lipid metabolic reprogramming in HPV-KI is characterized by decreased levels of FFA, PA CAR and S1P, but increased abundance of sphingolipids. The asymmetry in up-regulated sphingolipids and reduced secretion of S1P in FSG67-treated HPV-KI is in concordance with previous findings that knock-down of SPHK1 blocked S1P production and increased levels of sphingosine and ceramide, which are S1P precursors [35].

Given that inhibition of glycolysis and lipid biogenesis results in differential metabolic reprogramming, and might both have unexpected side effects due to the resulted profound changes in metablisms, we opt to target the downstream S1PRs to treat cervical cancer, the overall response rate of which is still low even with the cutting-edge immunotherapy. Several lines of evidence supports this approach. First, previous study showed that the SpHK1-mediated high expression of PD-L1 in melanoma promotes the immune resistance and that this dysfunction can be rescued by PD-1 blockade. In addition, SPHK1 axis holds the potential to predict melanoma patient outcomes in response to anti-PD-1 mAb therapies [36]. Similarly, the MP6 gene set which contains *S100A8/A9* downstream of SpHK1/S1P/S1PRs has the potential to serve as a predictive marker for cervical cancer response to immune checkpoint blockers. Second, according to the correlation analysis of TCGA database, significant relationship between *SPHK1* expression and *S100A8/A9* expression was observed in ACC, KICH and COAD (Supplementary Table 10). Third, our study revealed a significant decrease in MP6 gene set expression in both HPV-KI and HeLa cells following W146 (a S1PR inhibitor) treatment, indicating its potential use as an adjuvant therapy for cervical cancer. Indeed, targeting S1PR1 demonstrates significant potential in vitro and *vivo* for controlling tumor growth in this study. Moreover, treatment with W146 results in the down-regulation of *S100A8*/*A9* expression, as well as decreased S100A8/A9 protein levels in the PDX model. Therefore, these findings underscore the anti-tumor potential of W146 and suggest that S1PR1 may represent a promising therapeutic target for the prevention and treatment of cervical cancer.

In conclusion, this study has established the HPV18 knock-in cell model as a robust and reliable system for investigating the molecular mechanisms of cervical carcinogenesis. Multi-omics analysis reveals that metabolic reprogramming in HPV-KI cells regulates the expression of S100A8/A9 and activates the NF-κB/MAPK signaling pathways. The SpHK1/S1P/S1PR1 pathway, which is modulated by glycolytic and lipid metabolic reprogramming, may serve as a potential therapeutic target for cervical cancer treatment.

However, this study also comes with limitations. First, although elevated glycolysis has been widely observed in cervical cancer and other types of cancers, the mechanisms by which HPV-KI activates glycolysis is still unclear. Second, this study chooses E6 and E7, the two most important viral oncogenes, to investigate the biological consequence of HPV18 integration; however, the effect of other HPV early genes (i.e., E1, E2, E4, and E5) that also play important role in HPV infection and/or cervical carcinogenesis the primary oncoproteins, is overlooked, largely due to the technical limitations. With advancements in technology, it is now feasible to develop templates encompassing longer HPV genomic sequences, enabling the generation of diverse HPV gene knock-in cell lines. Therefore, further investigations are warranted to overcome these caveats, which will give more insights into the better understanding of the mechanisms by which HPV integration promotes the development of cervical cancer, thereby facilitating the development of more effective treatment strategies for this malignant disease.

## Materials and methods

### Plasmid construction and preparation

All sgRNAs were designed based on the hg19 genome using a website tool (https://tools.genome-engineering.org). The gene sequences around the HPV integration site were carefully selected, and we identified the three sgRNAs with the highest score and fewest off-target matches (gRNA1: ACATATACTTTCAAACATCATGG, gRNA2: ACAAATAACTGTAAAGAGTAAGG and gRNA3: AACAGAAAGTAAAGATCTTTAGG). They were cloned following the protocol from Feng Zhang lab, which involved separately ordering oligonucleotides complementary to gRNA templates, annealing them, phosphorylating them, and finally cloning them into the *BbsI* sites of pSpCas9(BB)-2A-EGFP (Addgene plasmid, Cambridge, MA, USA).

The sgRNA sequence used in the study was gRNA2: ACAAATAACTGTAAAGAGTAAGG. For the construction of donor vectors, 500 bp left and right homology arms were amplified from HaCaT genomic DNA by PCR and cloned into pDC515 to generate pDC515-HAL-HAR (D0). Subsequently, the flag-Loxp+CMV-EGFP-2A-Puro-polyA+Loxp cassette was generated and inserted downstream of the left arm of D0, resulting in pDC515-HAL-flag-Loxp+CMV-EGFP-2A-Puro-polyA+loxp-HAR(D1), which served as the control donor vector. To obtain HPV18 knock-in donor vectors, the HPV18 URR + E6 + E7 gene was cloned from HPV18 BAC, reversed, and inserted upstream of the EGFP cassette of D1 to generate pDC515-HAL-HPV18 E7 + E6 + URR-flag-Loxp+CMV-EGFP-2A-Puro-polyA+loxp-HAR (D2). All plasmids were prepared using an endotoxin-free plasmid extraction kit (D6915, Omega, USA) and stored at −80 °C.

### Cell culture and transfection

HaCaT cells, derived from a transformed nontumorigenic human keratinocyte cell line, and Human papillomavirus-related cervical cancer cell lines (HeLa) were obtained from ATCC. HaCaT and its derivative cell line HPV-KI were cultured in fresh 1640 median (1640; Invitrogen) supplemented with 15% fetal bovine serum (FBS, Gibco), while HeLa cells were cultured in Dulbecco’s minimum essential medium (DMEM; Invitrogen) with 10% FBS. Additionally, 100 U/mL penicillin/streptomycin (Invitrogen) were added to the media. All cells were grown in a humidified incubator at 37 °C in a 5% carbon dioxide atmosphere and transfected using linear polyethylenimine (PEI) (Sigma; 764604).

### Single cell cloning and analysis

After puromycin selection for 5 days, the cells were trypsinized and EGFP-positive cells were sorted into 96-well plates using BD-FACS under sterile conditions and cultured at 37 °C with 5% CO2. Each well was supplemented with 100 μL of fresh 1640 medium containing puromycin (25 mM) and refreshed weekly. After 3 weeks, the EGFP-positive cell colonies were transferred to 24-well plates.

### Flow cytometry and Fluorescence activated cell sorting

To assess the proportions of EGFP-positive cells, the cells were resuspended in PBS and fixed with 2% paraformaldehyde for 10 min, followed by flow cytometry analysis using a FACSAria II cell sorter (BD Biosciences). Isolation of EGFP-positive cells was also performed using FACSAria II cell sorter (BD Biosciences)

### Quantitative reverse transcription PCR (RT–PCR)

Total RNA was extracted using the Total RNA Kit I (OMEGA; R6834). Subsequently, RT-PCR was conducted with 2 μg of total RNA. Quantitative RT-PCR analysis was carried out using a CFX96 Real-Time system (Bio–Rad, USA). All experiments were performed in triplicate, and the primers for all target genes and ACTB (reference gene) can be found in Supplementary Table 1.

### Colony formation assay

A total of 500 cells were seeded in triplicate in 12-well plates and incubated for 2 weeks. The resulting colonies were stained with 0.04% crystal violet and photographed. Colonies larger than 50 μm in diameter were quantified using an Omnicon 3600 image analysis system.

### Cell proliferation assay

Cell proliferation was assessed using Cell Counting Kit-8 (Dojindo Molecular Technologies) following the manufacturer’s protocol. Briefly, 5 × 10^3^ cells were plated in 96-well plates and incubated for 0 h, 24 h, 48 h, 72 h, and 96 h in a 37 °C incubator with 5% CO2. Subsequently, CCK-8 dye was added and incubated at 37 °C for 3 h, followed by measuring the absorbance at 450 nm using a microplate reader.

### Transwell migration and invasion assay

Cells were diluted to a concentration of 5 × 10^5^ cells/ml in serum-free DMEM, and 100 μl of the cell suspension was added to Transwell chambers (Corning Life Sciences). Cells were then seeded into the upper compartment and incubated for 24 at 37 °C. The lower compartment was filled with medium supplemented with 20% FBS, and stained cells on the entire filter were counted under a microscope.

### Wound healing assay

The cells were cultured in six-well plates until reaching full confluence. A wound was created by scratching the monolayer with a 200 μl pipette tip once the cell density reached 100%. After washing the cells three times with 1× PBS to remove debris, they were incubated in fresh serum-free DMEM. The area of the scratch was photographed at 0 h, 12 h, and 24 h, and the distance from the scratch was measured at each time point. Finally, the wound healing effect was quantified using the following formula: 100 (scratch width at 0 h - scratch width at 24 h)/scratch width at 0 h. Each experiment was performed in triplicate for statistical analysis.

### Transcriptome sequencing and analysis

Total mRNA with polyA tail was extracted and reverse transcribed to cDNA for sequencing. Three biological replications were performed for each sample and were sequenced for each replicate by Illumina platform with PE150. The sequenced reads were mapped to the hg19-HPV reference genome (We inserted a 4284 bp HPV sequence at the chr8:128230430 site and reconstructed the new genome, which we called hg19-HPV genome below) using HISAT2 [37] with default parameters. We used HTSeq to evaluate reads count of each gene and FPKM (Fragments per Kilobase per Million Mapped Fragments) to quantify gene expression level. Differential gene expression analysis was used by DESeq2 R package with |log_2_ Foldchange | ≥1 and *q* < 0.05 (FDR-adjusted *p*-value) as a cutoff [38, 39]. The data of this study are available in the NCBI Gene Expression Omnibus (GEO) at https://www.ncbi.nlm.nih.gov/geo/, reference number GSE285710 and GSE285796.

### ChIP sequencing and analysis

We first harvested cells and centrifuged for 3 min at 600 × *g* at room temperature. Follwing step is wash the sample twice in Wash Buffer by gentle pipetting. Then cell nucleus was prepared and resuspended. Next phase if incubation of sample and primary antibody overnight at 4 °C on a rotating platform. After centrifuge, the primary antibody was removed carefully. To increase the number of Protein A binding sites for each bound antibody, we used secondary antibody which was diluted in DigWash buffer and to incubate cells for 30 min at RT. In next move, cells were washed using DigWash buffer to remove unbound antibodies. Dilution of pA - Tn5 adapter complex was prepared and added to the nucleus with gentle vortexing, Then, nucleus incubated at RT for 1 h and washed in wash Buffer to remove unbound pA - Tn5 protein. In the subsequent step, we resuspended nulcear in Tagmentation buffer and incubated for 1 h at 37 °C. For stop tagmentation, STOP buffer added to sample after incubation at 55 °C for 30 min, and then at 70 °C for 20 min. Library DNA was then purified and amplified for sequencing with the Illumina NovaSeq 6000. Details of antibodies used in ChIP can be found in Supplementary Table 21.

ChIP-seq reads were mapped to hg19-HPV reference genome by bowtie2 software, and only uniquely and non-duplicate mapped reads were used to perform the downstream analysis. We used deeptools to generate the correlation plot for all samples [40]. Peak calling was called by using MACS2 [41], and following step is annotation of peaks by using bedtools. Reads coverage and depth were calculated by samtools. MAnorm software were tried to define differential H3K27ac peaks between two groups [14]. Read count data was performed by converting raw bam files to bigwig files and then as input data sets of deeptools to plot heat-maps and profiles.

### In nucleus Hi-C experiments

About one million cells were crosslinked by 40 ml 1% formaldehyde solution at room temperature for 10 min and 2.5 M Glycine was added to quench the crosslinking reaction. Supernatant was removed and the cells were washed three times by 1×PBS. Crosslinked cells were resuspended in 500 µL of ice-cold Hi-C lysis buffer and rotated at 4 °C for 30 min. The nuclei were washed by 0.5 ml of restriction enzyme buffer and the chromatin is solubilized with dilute SDS. After quenching the SDS by Triton X-100, overnight digestion was applied with cutter restriction enzymes (400 units MboI) at 37 °C. The DNA ends were marked with biotin-14-dCTP and performed blunt-end ligation of crosslinked fragments. The proximal chromatin DNA was re-ligated by ligation enzyme and the nuclear complexes were reversed crosslinked at 65 °C. DNA was purified and Biotin-C was removed from non-ligated fragment ends using T4 DNA polymerase. Fragments was sheared to a size of 100–500 base pairs by sonication. The fragment ends were repaired by the mixture of T4 DNA polymerase, T4 polynucleotide kinase and Klenow DNA polymerase. Biotin labeled Hi-C sample were specifically enriched using streptavidin magnetic beads. The fragment ends were adding A-tailing by Klenow (exo-) and then adding Illumina paired-end sequencing adapter by ligation mix. At last, the Hi-C libraries were amplified by 8–10 cycles PCR, and sequenced in Illumina HiSeq instrument with 2 × 150 bp reads.

### Hi-C data processing

Mapping, filtering, correction and generation of Hi–C matrices were done using HiC-Pro (https://github.com/nservant/HiC-Pro). Briefly, read pairs were mapped independently to hg19-HPV reference genome using Bowtie2 (end-to-end algorithm and “-very-sensitive” option) with HiC-Pro (V2.7.8) default parameters. After filtering, valid-pairs were used to generate raw and ICE (Iterative correction and eigenvector decomposition) normalized matrices at 20 kb, 40 kb, 100 kb and 1 M bin resolution. The ICE matrices are normalized matrices which refers to the iterative correction method to correct the biases such as GC content, capability and effective fragment length in Hi-C data. On the other hand, we built the hic format file for each sample with 2500000, 1000000, 500000, 250000, 100000, 50000, 25000, 10000, 5000,1000 resolutions by using the juicer tool from valid pairs reads, then transformed them to mcool format with hicConvertFormat tool [42].

### TAD signal variance analysis

TAD signal variance can indicate the strength of the TAD structure and we calculated using scripts of the analysis in Chen et al. [12] (https://github.com/ChenXP0310/2019-humanembryo3D/blob/master/scripts/). We used intra-chromosomal maps at 20-kb resolution with iced matrix. In brief, TAD signal was calculated as the log_2_ ratio of the number of corrected upstream-to-downstream interactions within a 2-Mb window in each chromosome. Regions which contained less than 10 counts or gaps within a 2-Mb distance were filtered out. The variance was calculated for individual chromosomes. The Wilcoxon rank-sum test was applied to test statistical significance [12]. The Hi-C and ChIP-seq data are available at https://www.ncbi.nlm.nih.gov/bioproject/, reference number PRJNA1206105.

### Multiomics statistical analysis

The relationship between interaction changes were used *Fisher*’s test and wilcoxon rank sum test to determine, transcriptome and epigenome signals. Annoroad-OMIC-Viz (https://github.com/Spartanzhao/Annoroad-OMIC-Viz) and trackc (https://github.com/seqyuan/trackC) were used to generate multiomics display figures.

### KEGG pathway functional analysis

The KEGG pathway (https://www.genome.jp/kegg/pathway.html) enrichment of DEGs was implemented by the hyper geometric test (*Fisher*’s exact test) by using enrichKEGG function and GSEA analysis was performed with gseKEGG function in clusterProfiler R package, in which *p*-value is calculated and adjusted as *q*-value. KEGG terms with *q* < 0.05 were considered to be significantly enriched [43].

### K14-HPV16 transgenic mice

K14-HPV16 transgenic mice have been described previously [44]. Breeding pairs of K14-HPV16 transgenic mice were provided by the National Cancer Institute (NCI) Mouse Repository (Frederick, Maryland, USA) [strain nomenclature: FVB.Cg-Tg (KRT14-HPV16) wt1Dh] and bred at the Experimental Animal Center, Tongji Medical College, HUST. All animal care and experimental procedures strictly adhered to the guidelines for ethical review of animal welfare and were granted approval by the Institutional Animal Care and Use Committee at Huazhong University of Science and Technology (TJH-202001007). The RNA-seq data of K14-HPV16 and FVB mice are available in the Genome Sequence Archive (GSA) at https://ngdc.cncb.ac.cn/gsa/, reference number CRA005449.

### Immunohistochemistry (IHC) and scoring

The tissue sections were initially processed according to the standard procedure. Antigen retrieval was subsequently performed using 1 mM EDTA (pH 9.0; G1203, Servicebio, Wuhan, China) for Ki-67 and 10 mM citric acid-sodium citrate buffer (pH 6.0; G1202, Servicebio, Wuhan, China) for S100A8/A9. The sections were then sequentially incubated in 3% hydrogen peroxide to block endogenous peroxidase activity and in 5% bovine serum albumin (BSA; G5001, Servicebio, Wuhan, China) to neutralize non-specific binding. Following this, the sections were incubated overnight at 4 °C with diluted primary antibodies. Detailed antibody information is provided in Supplementary Table 21. Subsequently, the sections were treated with an HRP-conjugated secondary antibody (AS014, Abclonal, Wuhan, China), and immunostaining was visualized using diaminobenzidine (DAB; G1211, Servicebio, Wuhan, China). Finally, the sections were counterstained, dehydrated and mounted.

Immunohistochemical evaluation was conducted independently of the H&E diagnosis by two experienced pathologists. In cervical cancer tissues, S100A8/A9 expression in tumor cells was evaluated based on staining intensity, which was categorized as follows: no staining (0), weak staining (1 + ), moderate staining (2 + ), and strong staining (3 + ). A semi-quantitative histologic scoring system (HSCORE) was applied, calculated using the formula: HSCORE = [1 × (% cells stained 1 + ) + 2 × (% cells stained 2 + ) + 3 × (% cells stained 3 + )], yielding a score ranging from 0 to 300. Additionally, the percentage of Ki-67-positive (Ki-67⁺) cells was determined to assess the proliferation rate in the patient-derived xenograft (PDX) model.

### Protein-protein interaction

The protein-protein interaction (PPI) networks for IL-17 signaling pathway, TNF signaling pathway, NF-kappaB signaling pathway and MAPK signaling pathway were constructed with the STRING database and visualized with Cytoscape (version 3.7.1)

### S1P secretion ELISA assay

10^6^ cells were cultured in six-well plates and incubated with complete 1640 medium with or without drugs for 24 h. The cells were then washed with PBS and incubated with serum-free 1640 medium for an additional 12 h. Following this, the supernatant was collected and detected according to the manufacturer’s instructions (Cat No#KBH1860, KRISHGEN BioSystems, INDIA).

### Metabolomics analysis of the cells

10^7^ cells were cultured or treated for 24 h. After removing the medium, ice-cold PBS was quickly used to wash the cells for 2–3 times. After gently scratching the cells with 2–3 ml ice-cold PBS, the PBS was transfer to a centrifuge tube, centrifuged at 300–500g for 5 min at 4 °C. discard the supernatant, wash with ice-cold PBS solution at 4 °C, and remove the PBS. The cells were collected, frozen in liquid nitrogen for 5–10 min, the samples were stored at −80 °C.

Extraction and detection for Energy metabolome assay and quantitative lipid-omics were performed by Metware Biotechnology Co., Ltd. (Wuhan, China).

All those metabolites were detected by MetWare (http://www.metware.cn/) based on the AB Sciex QTRAP 6500 LC-MS/MS platform.

### Differential metabolites selected

For two-group analysis, differential metabolites were determined by VIP (VIP > 1) and *P*-value (*P*-value < 0.05, Student’s *t* test). VIP values were extracted from OPLS-DA result, which also contain score plots and permutation plots, was generated using R package MetaboAnalystR. The data was log_2_ and mean centering before OPLS-DA. In order to avoid overfitting, a permutation test (200 permutations) was performed.

### KEGG annotation and enrichment analysis for energy metabolome assay and quantitative lipid-omics

Identified metabolites were annotated using KEGG Compound database (http://www.kegg.jp/kegg/compound/), annotated metabolites were then mapped to KEGG Pathway database (http://www.kegg.jp/kegg/pathway.html). Pathways with significantly regulated metabolites mapped to were then fed into MSEA (metabolite sets enrichment analysis), their significance was determined by hypergeometric test’s *p*-values.

### DA score

Differential Abundance Score (DA Score) is a pathway-based metabolic change analysis method. The Score can capture the overall change of all metabolites in a certain pathway. The DA Score is calculated by: Number of up-regulated differential metabolites-number of down-regulated differential metabolites) /number of all metabolites annotated to this pathway. DA score is with a ranking between −1 and 1.

### Enzyme activity detection

10^6^ Cells were seeded and cultured in a 6-well plate, cells were treated with drugs or seeded 24 h prior to the assay. The activity of GPDH, SpHK1/2, HK, PFK and PK was measured using an assay kit according to the manufacturer’s instructions. Briefly, cells were washed with ice-cold PBS and then extracted with provided extraction buffer through freeze/thaw cycles. Following centrifugation, enzyme activity was determined in the supernatants. the kits used in the study were: Glycerol-3-Phosphate Dehydrogenase (G3PDH) Assay Kit (Colorimetric) (ab174095, abcam, USA), Sphingosine Kinase Activity Assay (K-3500, Echelon Biosciences Inc., USA), CheKine™ Micro Hexokinase (HK) Activity Assay Kit (KTB1123, abbkine, China), CheKine™ Micro 6-Phosphofructo kinase (PFK) Activity Assay Kit (KTB1124, abbkine, China) and CheKine™ Micro Pyruvate Kinase (PK) Assay Kit (KTB1120, abbkine, China).

### Phosphatidic acid quantification

Phosphatidic acid quantification was conducted using the Phosphatidic Acid Assay Kit (Fluorometric, ab273335, abcam, USA). 10^6^ cells were seeded and cultured in a 6-well plate, cells were treated with drugs or seeded 24 h prior to the assay, and the lipid extraction and quantification procedure were performed following the manufacturer’s instructions.

### Western blot assay

The proteins were isolated and separated using Sodium dodecyl sulfate–polyacrylamide gel electrophoresis (SDS-PAGE), then transferred to a polyvinylidene difluoride membrane (PVDF) and incubated with primary antibodies for 12 h at 4 °C. Protein visualization was achieved using the ECL system (Bio–Rad) and analysis was performed using Image Lab (6.0.1). The specific antibodies and their dilutions used are listed in Supplementary Table 21. The unedited complete Western blot images were uploaded in the attached materials.

### Patients and tissue samples

We recruited 108 CC patients admitted in Tongji Hospital of Huazhong University of Science and Technology between June 2018 and January 2021. All patients provided written informed consent. The research was approved by the Ethical Committee of Tongji Medical College, Huazhong University of Science and Technology (TJ-IRB20180505).

### Animals

Female nonobese diabetic/severe combined immunodeficiency (NOD/SCID) mice, aged five to eight weeks and weighed 18–21 g, were purchased from Gempharmatech Co., Ltd (Nanjing, China). The experimental mice were housed in isolator cages maintained under specific-pathogen free conditions, with precisely regulated temperature and humidity, and with a standardized 12 h light/dark cycle at Tongji Medical College. All animal care and experimental procedures strictly adhered to the guidelines for ethical review of animal welfare and were granted approval by the Institutional Animal Care and Use Committee at Huazhong University of Science and Technology (lACUC Number: 2825).

### Establishment of PDX models

The PDX models were established following the protocol outlined by Liu et al. [45]. Upon reaching a size of 1500 mm^3^, the tumor from the P4 generation of patient CAT079 was harvested and used for implantation. Subsequently, tumor fragments measured 3–4 mm^3^ were then subcutaneously implanted into the flanks of NOD/SCID mice to initiate the PDX experiment.

### W146/JTE013 treatment in vivo

The treatment was initiated when the average tumor volumes ranging from 30 to 50 mm^3^ across all experimental group. The NOD/SCID mice in the W146 treatment group were treated with either phosphate-buffered saline (PBS) or 5 mg/kg of W146 administered via intraperitoneal injection every other day since the tumor size reached 30 mm^3^. After 18 days of treatment, tumors were harvested and analyzed. In the JTE013 group, NOD-SCID mice bearing PDX tumors were administered 15 mg/kg JTE013 by gavage every 3 days, with DMSO/TWEEN-20/PEG used as control. The tumor volumes and body weight of mice were measured every 3 days. Tumor volume was calculated using the formula: tumor volume = 0.52 × length (L) × width (W)^2^.

## Supplementary information

Supplementary Tables 1–21

Supplementary Figure 1–11

Western blot unedited-revised
