# Supplementary material for: Site-specific HPV18 integration facilitates cervical carcinogenesis through metabolic reprogramming-induced dysfunction of the SpHK1/S1P/S1PR1 pathway
Source: Cell Death Dis. 2026 Jan 9;17(1):24. doi: 10.1038/s41419-025-08195-7 (PMC12789681; doi:10.1038/s41419-025-08195-7)
Supplement: Supplementary file 2 — Supplementary Figure 1–11 [file 41419_2025_8195_MOESM2_ESM.pdf]

Supplementary Figures

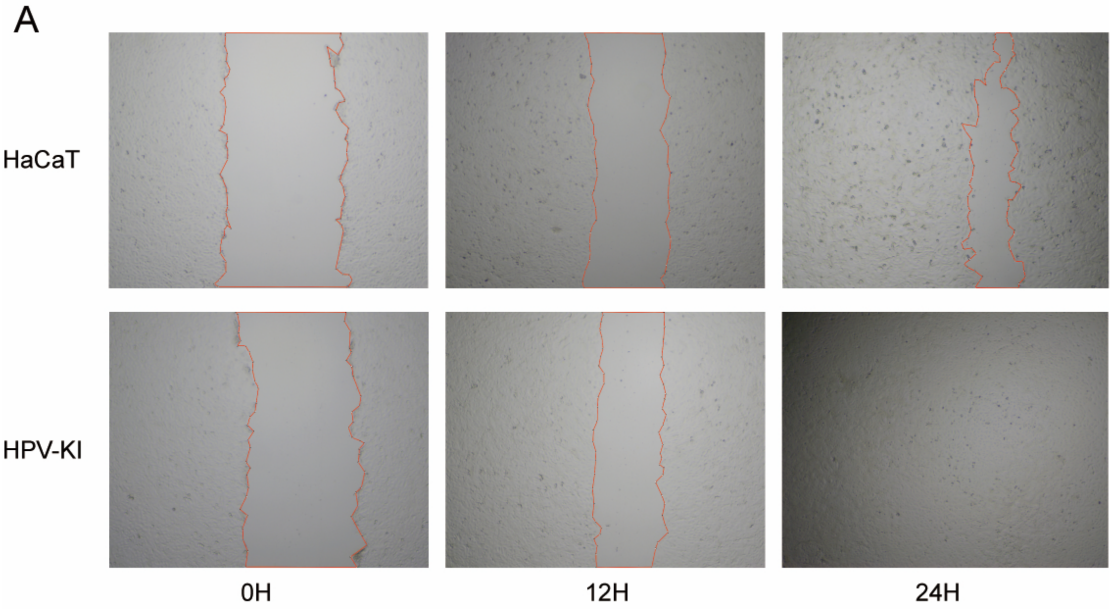

Supplementary Figure 1. (A) Wound healing assay of HaCaT and HPV-KI at 0h, 12h and 24h. wound healing rates are shown in **Fig. 1G**.

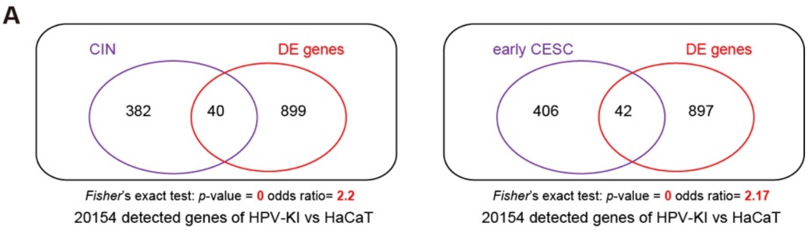

Supplementary Figure 2. (A) The left panel presents a *Fisher*'s exact test comparing DE genes between HPV-KI and HaCaT with those from scRNA data of CIN compared to normal epithelial cells. The right panel illustrates the comparison of DE genes between HPV-KI and HaCaT with those of early-stage CESC compared to normal epithelial cells. the DE gene list is from Liu *et al.*, Sci. Adv. 9, eadd8977(2023).

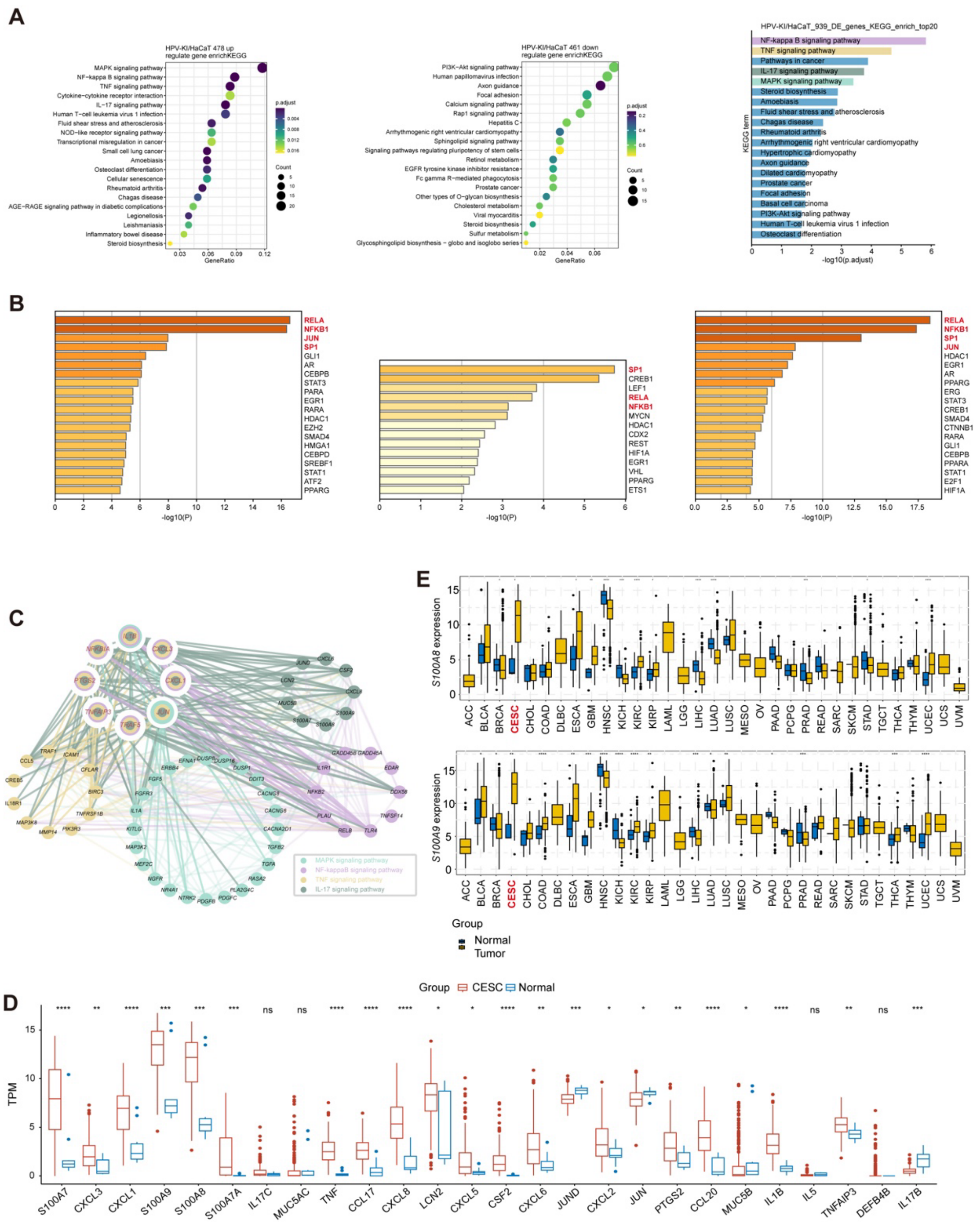

Supplementary Figure 3. (A) KEGG pathway enrichment analysis was performed for the up-regulated and down-regulated DE genes in HPV-KI/HaCaT cells. The top 20 enriched KEGG pathways were identified for 939 DEGs in HPV-KI/HaCaT cells. **(B)** The Metascape database was utilized to predict transcription factors associated with both the up-regulated and down-regulated DEGs, as well as the total set of 939 DEGs identified in HPV-KI/HaCaT cells. **(C)** A protein-protein interaction (PPI) network was constructed for important DEGs involved in the IL-17 signaling pathway, TNF signaling pathway, NF-κB signaling pathway and MAPK signaling pathway. Genes shared by at least two pathways were considered as "Hub Genes".

(D) The expression of IL-17 pathway related genes between CSCE and normal was compared using data from the TCGA and GTEx database. **(E)** The expression levels of *S100A8* and *S100A9* were compared between different types of cancers and control samples. The Wilcoxon rank-sum test was applied to test statistical significance.

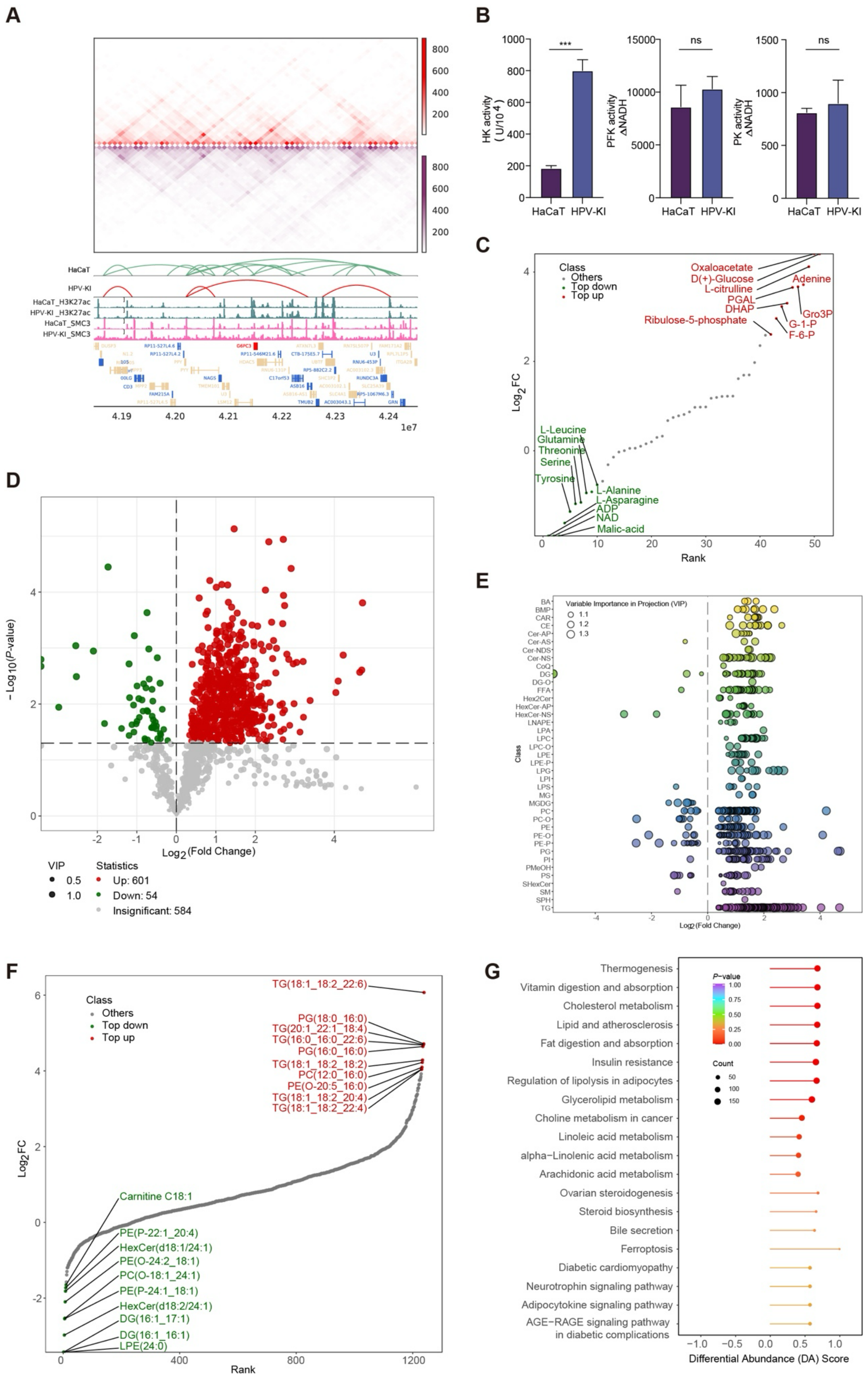

Supplementary Figure 4. (A) A Hi-C contact heatmap at the neighboring regions of *G6PC3*

(chr17: 42148097 - 42153712) along with corresponding tracks of the ChIP-seq profile were displayed. The gene track is shown in the bottom panel. **(B)** Bar plots illustrating the activity of HK, PFK, and PK in HPV-KI/HaCaT cells related to Fig. 4D. The data were calculated by two-tailed Student's *t*-test. *P* value was denoted as $^{***}P < 0.001$ and "ns" represented "not significant". **(C)** Dynamic distribution map of Fold change (FC) ranking of the difference in metabolite of carbon metabolism from HaCaT/HPV-KI. Top 10 metabolites down (green) and up (red) were marked. **(D)** Volcano plots were used to visualize the differences in the abundance levels of lipid metabolites in HaCaT/HPV-KI, with red spots indicating up-regulated differential metabolites, green spots indicating down-regulated differential metabolites, and grey spots indicating detected metabolites that did not show significant differences at $p < 0.05$. **(E)** Scatter plots were employed to illustrate the abundance differences of lipids from different subclasses in HaCaT/HPV-KI, with the abscissa representing the $\log_2$ FC between the two groups and the size of each dot representing its variable importance in projection (VIP) value. **(F)** Dynamic distribution map of FC ranking of the difference in metabolite in lipid-omics from HaCaT/HPV-KI. Top10 metabolites down (green) and up (red) were marked. (**G**) KEGG analysis reveals Lipid-omics changes between HPV-KI and HaCaT based on the DA Score of the pathway.

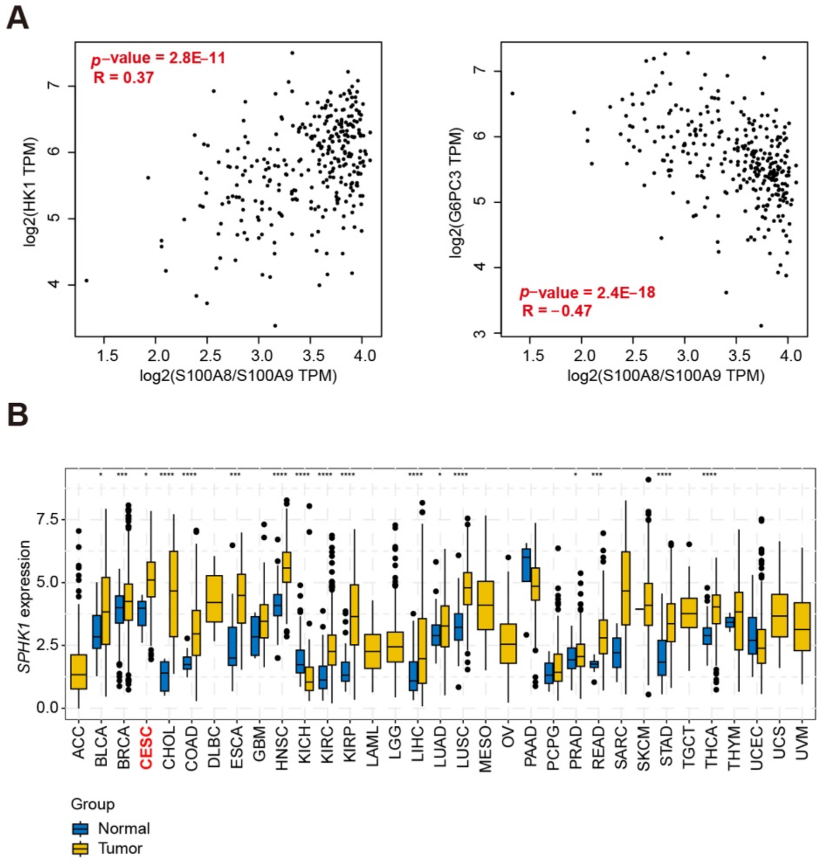

Supplementary Figure 5. (A) Correlation analysis of *S100A8/A9* with *HK1* and *G6PC3* in the TCGA database was conducted, and statistical analysis was performed using *Pearson's* correlation test. **(B)** Expression levels of *SPHK1* between different types of cancer and control respectively. The Wilcoxon rank-sum test was employed to access statistical significance.

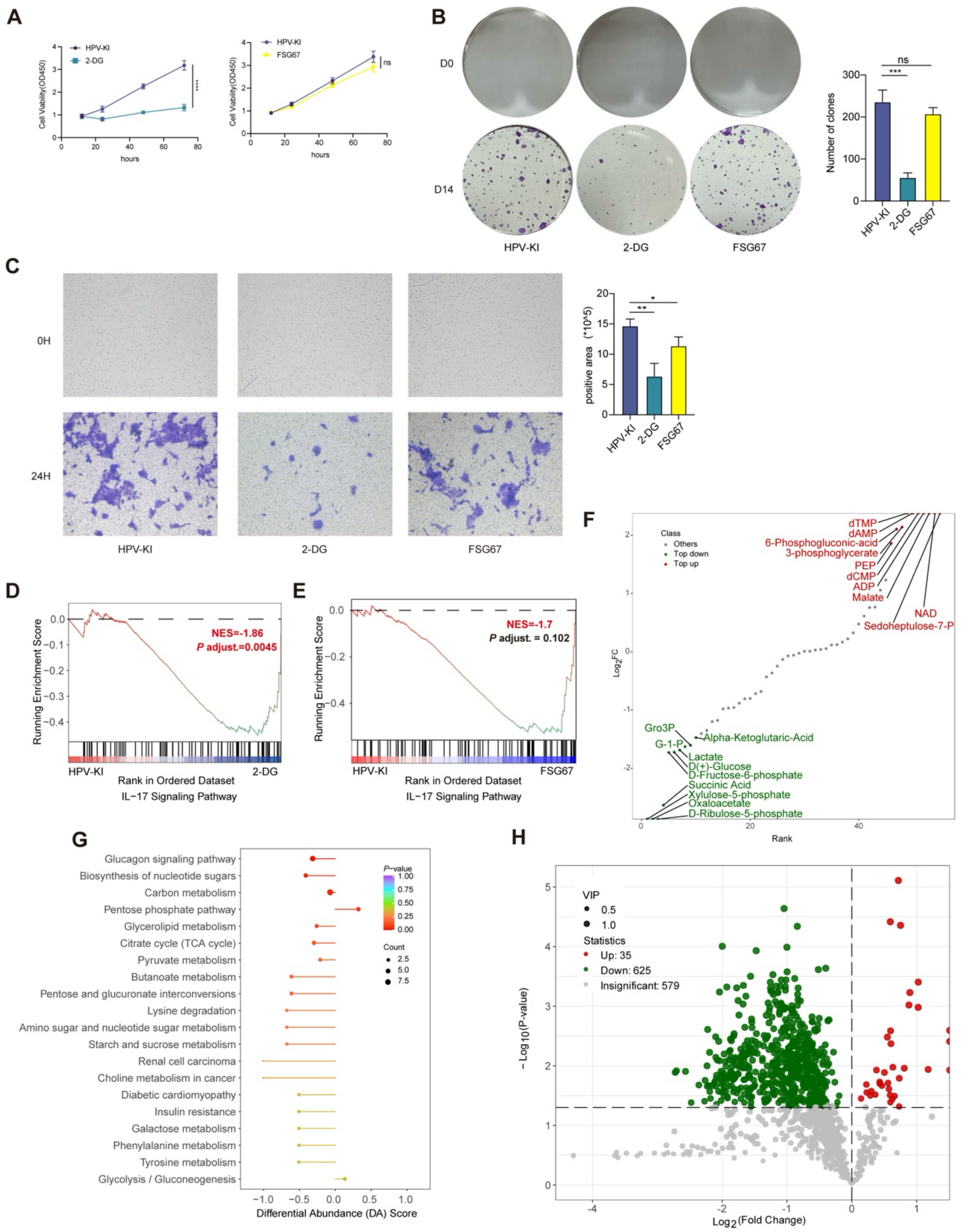

Supplementary Figure 6. (A) HPV-KI cell viability treated by 2-DG (2mmol) and FSG67 (20μmol) was detected by CCK-8. **(B)** Clone formation assay of HPV-KI, 2-DG- HPV-KI and FSG67- treated HPV-KI. **(C)** Transwell showing invasiveness of HPV-KI, 2-DG- treated HPV-KI and FSG67- treated HPV-KI. **(D)** GSEA analysis for IL-17 signaling pathway in 2-DG-treated HPV-KI/HPV-KI cells. (**E)** GSEA analysis was performed to investigate the IL-17 signaling pathway in FSG67- treated HPV-KI/HPV-KI cells. **(F)** Dynamic distribution map of

FC ranking of the difference in metabolites in carbon metabolism from 2-DG- treated HPV-KI /HPV-KI. Top10 metabolites down (green) and up (red) were marked. **(G)** KEGG analysis changes between 2-DG- treated HPV-KI and HPV-KI based on DA score in carbon metabolism. **(H)** Volcano plot showing the differences in the levels of lipid metabolites in 2-DG- treated HPV-KI /HPV-KI, with red spots indicating up-regulated differential metabolites, green spots indicating down-regulated differential metabolites, and grey spots indicating detected metabolites that did not show significant differences at $p < 0.05$.

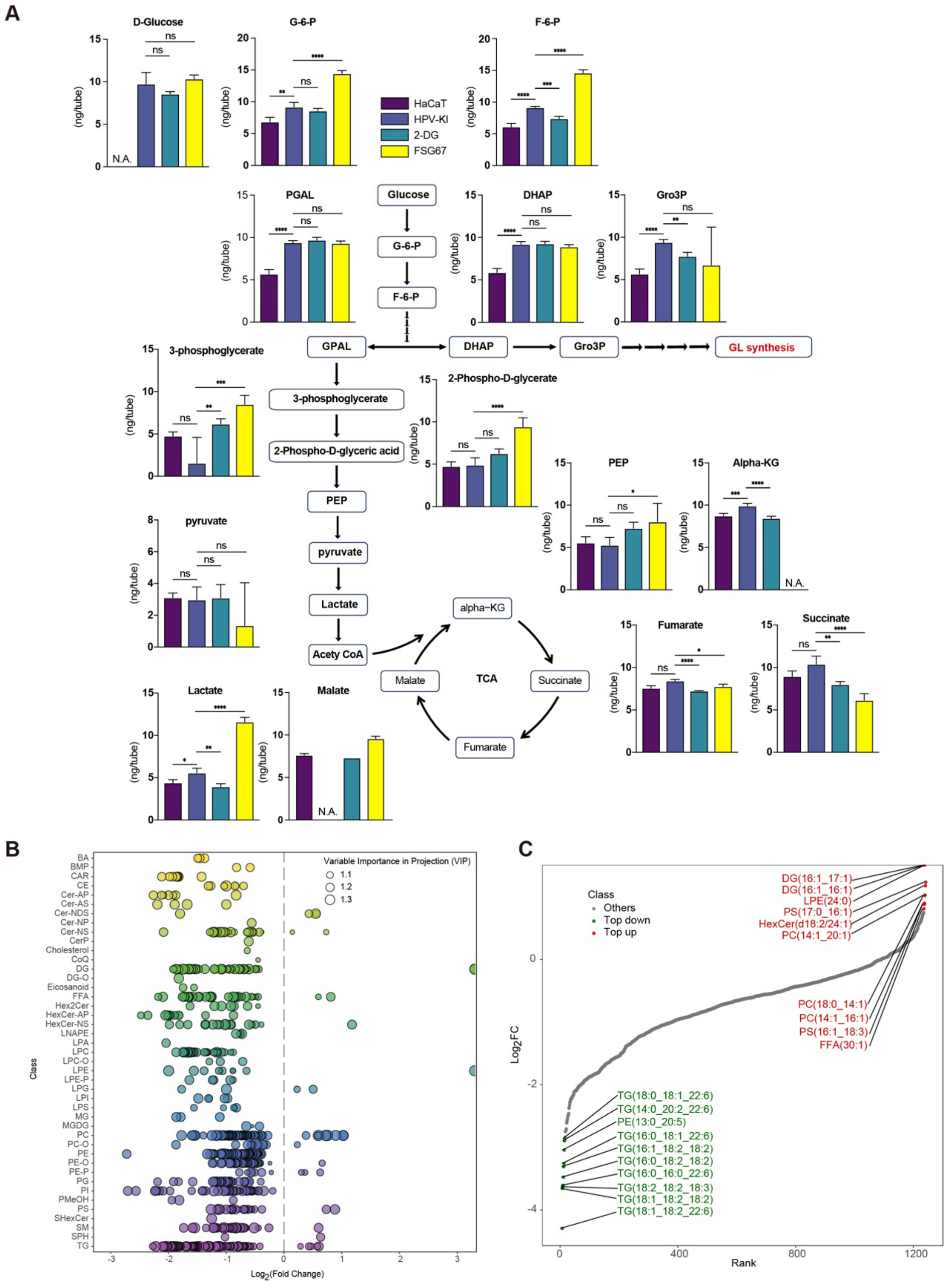

Supplementary Figure 7. (A) The abundance of metabolites in glycolysis and TCA cycle was compared among HaCaT, HPV-KI, 2-DG- treated HPV-KI and FSG67- treated HPV-KI using bar plots to display $\log_2$ FC values (one-way ANOVA-test, *$P < 0.05$, **$P < 0.01$, ***$P < 0.001$ and ****$P < 0.0001$, ‘‘ns” represented “not significant”, n/a indicates “not available”). **(B)** Scatter plot showing the abundance differences of lipids from different subclass in 2-DG-

treated HPV-KI /HPV-KI. **(C)** Dynamic distribution map of FC Ranking of the difference in metabolite in lipid-omics from 2-DG- treated HPV-KI /HPV-KI. Top10 metabolites down (green) and up (red) were marked.

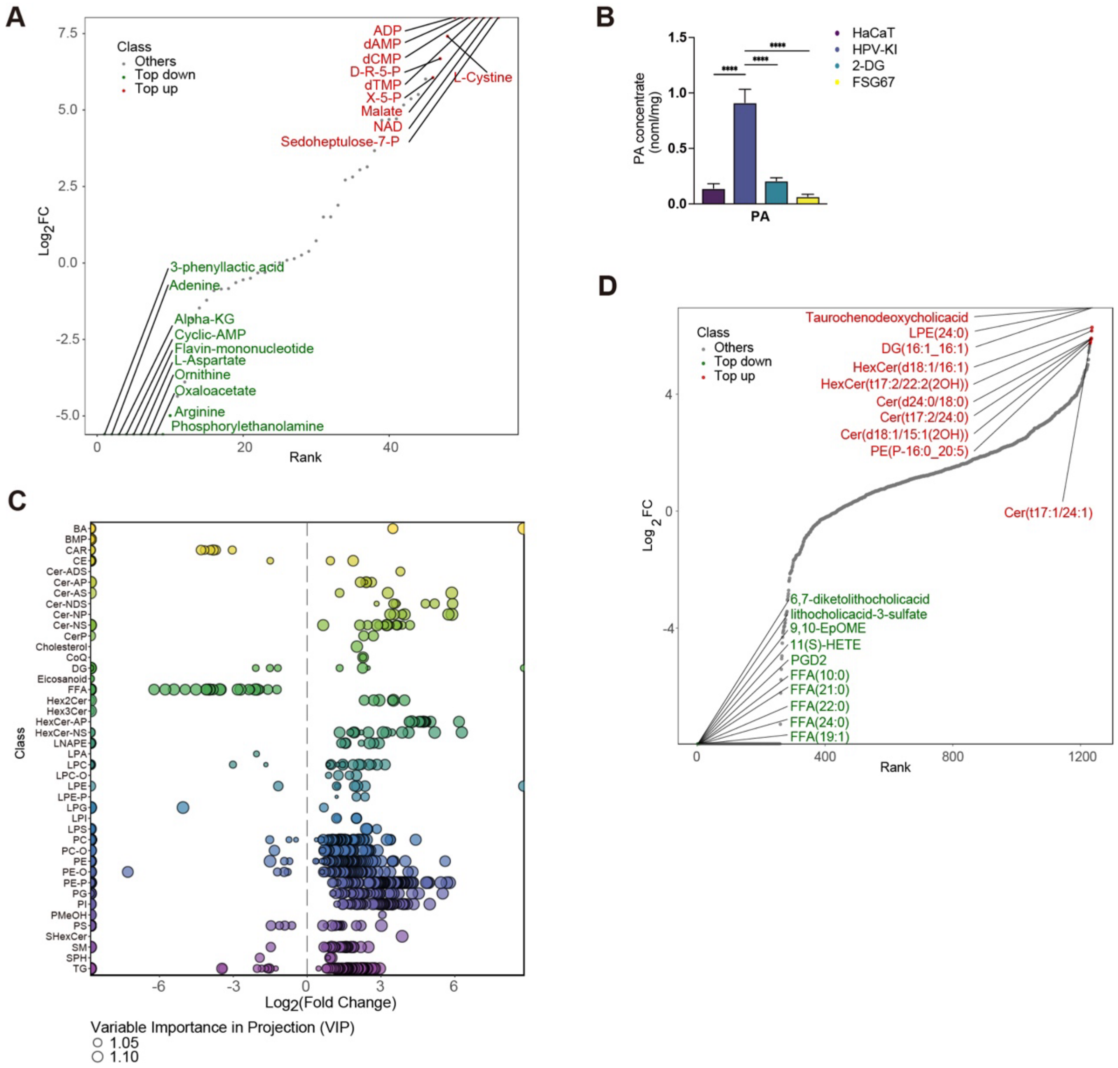

Supplementary Figure 8. (A) Dynamic distribution map of FC Ranking of the difference in metabolite in carbon metabolism from FSG67- treated HPV-KI /HPV-KI. Top10 metabolites down (green) and up (red) were marked. **(B)** The abundance of PA concentrate in HaCaT, HPV-KI, 2-DG- treated HPV-KI, and FSG67- treated HPV-KI is shown. **(C)** Scatter plot showing the abundance differences of lipids from different subclass in FSG67- treated HPV-KI /HPV-KI. **(D)** Dynamic distribution map of FC ranking of the difference in metabolite in lipid-omics from FSG67- treated HPV-KI/HPV-KI. Top10 metabolites down (green) and up (red) were marked.

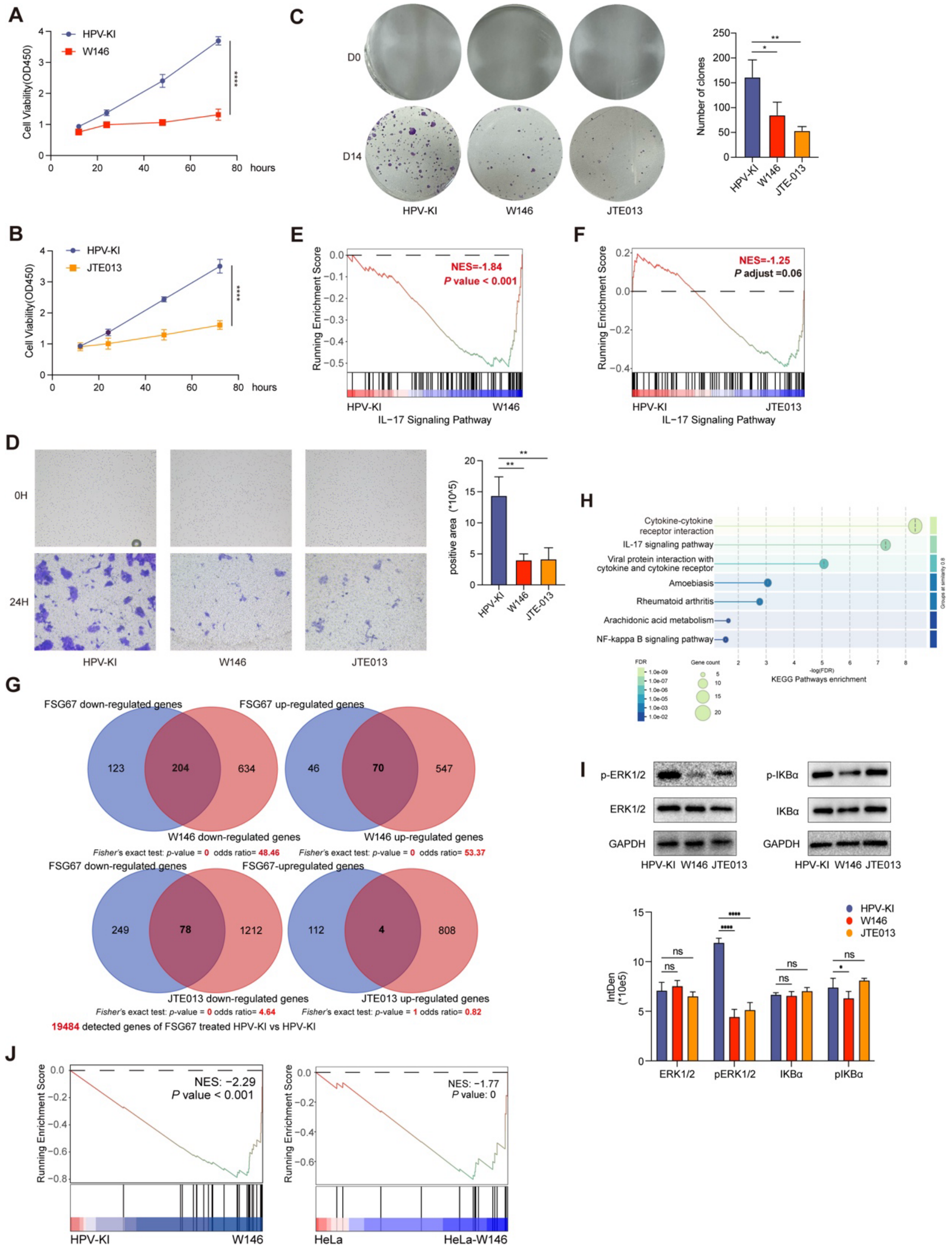

Supplementary Figure 9. (A) Cell viability of W146-treated and (**B)** JTE013-treated HPV-KI and untreated HPV-KI at 12 h, 24 h, 48h and 72 h has determined by CCK-8 assay. **(C)** Clone formation assay of HPV-KI, W146- treated HPV-KI and JTE013- treated HPV-KI. **(D)** Transwell showing invasiveness of HPV-KI, W146- treated HPV-KI and JTE013- treated HPV-KI. **(E)** GSEA analysis for IL-17 pathway in W146- treated HPV-KI/HPV-KI cells. **(F)** GSEA analysis for IL-17 pathway in JTE013- treated HPV-KI /HPV-KI cells. **(G)** Venn diagram

illustrating the up-regulation and down-regulation of genes between FSG67- and W146-, FSG67- and JTE013- treated HPV-KI. **(H)** KEGG enrichment for genes synchronically down-regulated in FSG67- and W146- treated HPV-KI. **(I)** ERK1/2, IκBα, phosphorylated ERK1/2, and phosphorylated IκBα protein levels in HPV-KI, W146- treated HPV-KI and JTE013-treated HPV-KI. The data were calculated by two-tailed Student's *t*-test. *P* value was denoted as $^*P < 0.05$, $^{**}P < 0.01$, and $^{***}P < 0.001$, $^{****}P < 0.0001$, "ns" represented "not significant".

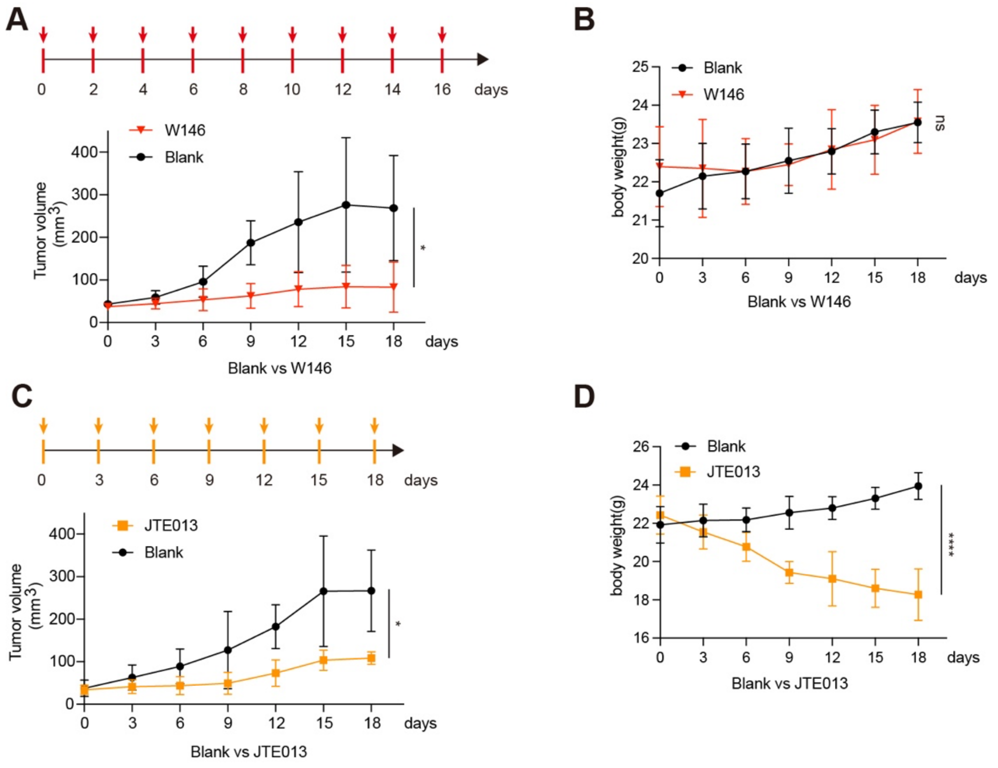

Supplementary Figure 10. (A) Tumor volumes of PDX tumors treated with PBS or W146, calculated using the formula 0.52×length (L)×width $(W)^2$. The arrow indicates the time of drug administration. **(B)** Body weight changes of PBS and W146- treated NOD/SCID mice. **(C)** Measurement of tumor volumes in PDX models following treatment with DMSO/TWEEN-20/PEG or JTE013. The arrow indicates the time of drug administration. **(D)** Body weight changes of DMSO/TWEEN-20/PEG and JTE013- treated NOD/SCID mice.

A

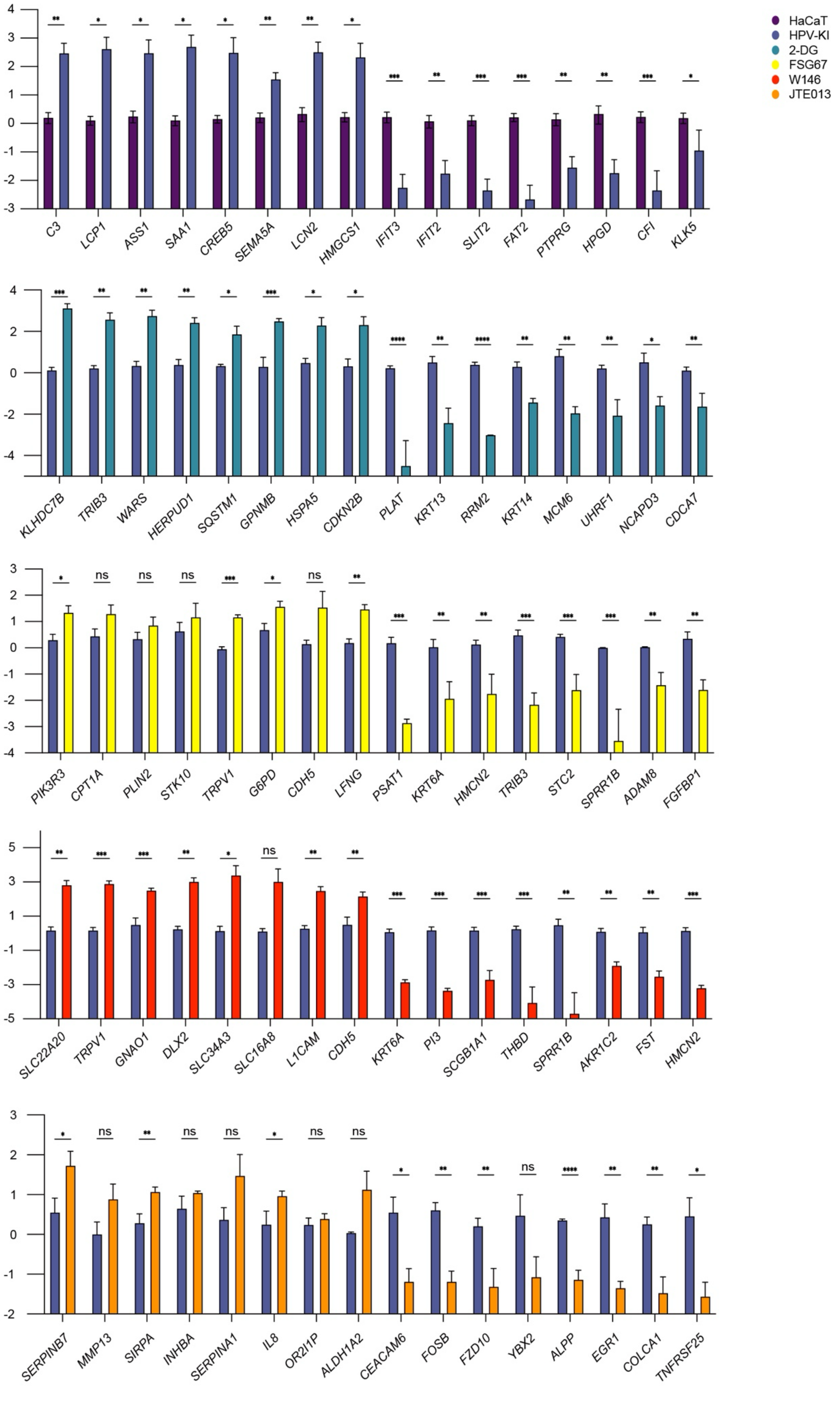

HaCaT
HPV-KI
2-DG
FSG67
W146
JTE013
C3
LCP1
ASS1
SAA1
CREB5
SEMA5A
LCN2
HMGCS1
IFIT3
IFIT2
SLIT2
FAT2
PTPRG
HPGD
CFI
KLK5
KLHDC7B
TRIB3
WARS
HERPUD1
SQSTM1
GPNMB
HSPA5
CDKN2B
PLAT
KRT13
RRM2
KRT14
MCM6
UHRF1
NCAPD3
CDCA7
PIK3R3
CPT1A
PLIN2
STK10
TRPV1
G6PD
CDH5
LFNG
PSAT1
KRT6A
HMCN2
TRIB3
STC2
SPRR1B
ADAM8
FGFBP1
SLC22A20
TRPV1
GNAO1
DLX2
SLC34A3
SLC16A8
L1CAM
CDH5
KRT6A
PI3
SCGB1A1
THBD
SPRR1B
AKR1C2
FST
HMCN2
SERPINB7
MMP13
SIRPA
INHBA
SERPINA1
IL8
OR2I1P
ALDH1A2
CEACAM6
FOSB
FZD10
YBX2
ALPP
EGR1
COLCA1
TNFRSF25

Supplementary Figure 11. (A) Validation of the top DEGs across experimental groups was conducted by qPCR, with $\log_2$ FC values calculated to quantify gene expression alterations. The primer sequences used for qPCR analysis are provided in Supplementary Table 1. The data were calculated by two-tailed Student's *t*-test. *P* value was denoted as *$P < 0.05$, **$P < 0.01$, and ***$P < 0.001$, ****$P < 0.0001$, "ns" represented "not significant".
