# Supplementary material for: Site-specific HPV18 integration facilitates cervical carcinogenesis through metabolic reprogramming-induced dysfunction of the SpHK1/S1P/S1PR1 pathway
Source: Cell Death Dis. 2026 Jan 9;17(1):24. doi: 10.1038/s41419-025-08195-7 (PMC12789681; doi:10.1038/s41419-025-08195-7)

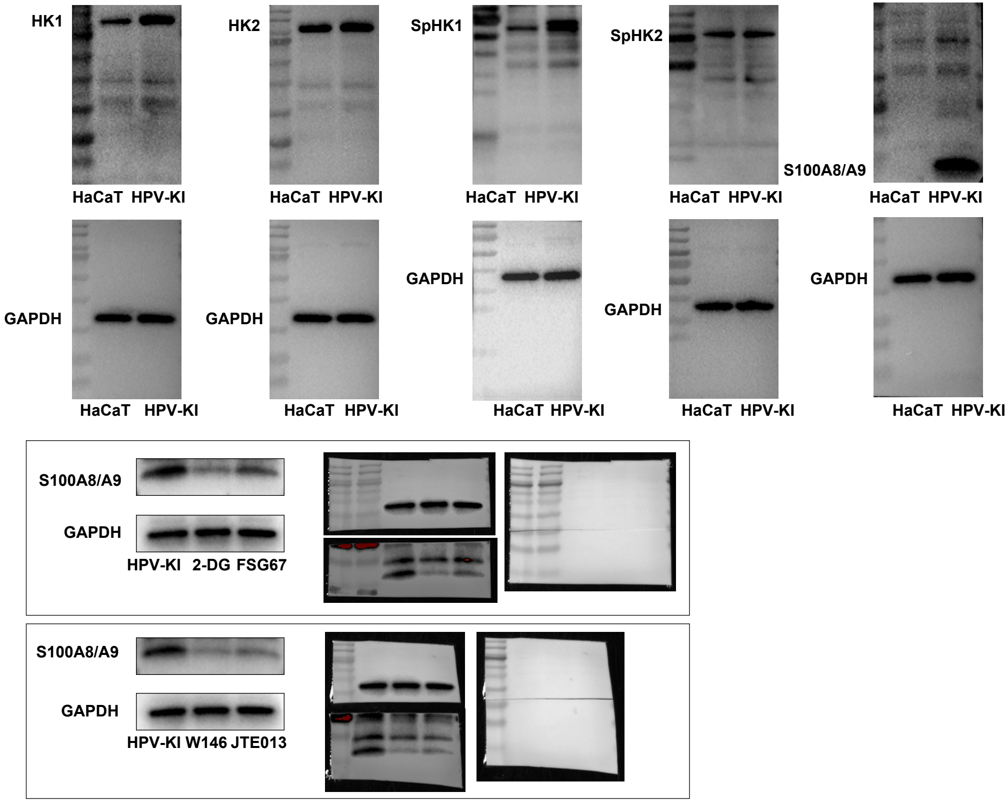
HK1
HK2
SpHK1
SpHK2
S100A8/A9
HaCaT HPV-KI
HaCaT HPV-KI
HaCaT HPV-KI
HaCaT HPV-KI
HaCaT HPV-KI
GAPDH
GAPDH
GAPDH
GAPDH
GAPDH
HaCaT HPV-KI
HaCaT HPV-KI
HaCaT HPV-KI
HaCaT HPV-KI
HaCaT HPV-KI
S100A8/A9
GAPDH
HPV-KI 2-DG FSG67
S100A8/A9
GAPDH
HPV-KI W146 JTE013

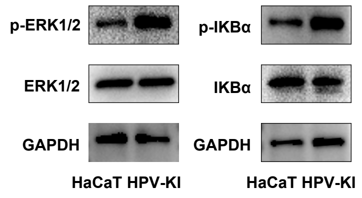

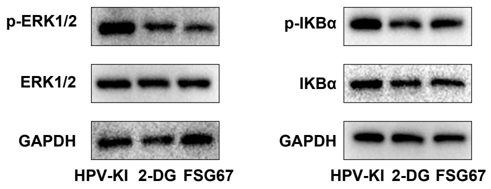

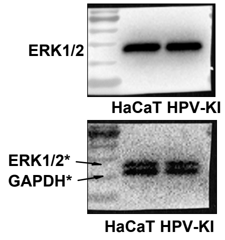

*Due to the high sensitivity of the ERK1/2 antibody, the primary/secondary antibodies could not be completely removed during the stripping process. Consequently, two distinct bands were observed when detecting the internal reference. These bands correspond to ERK1/2 and GAPDH, respectively.

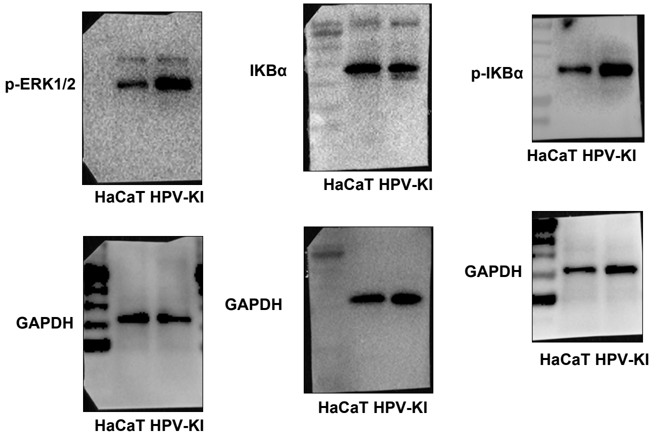

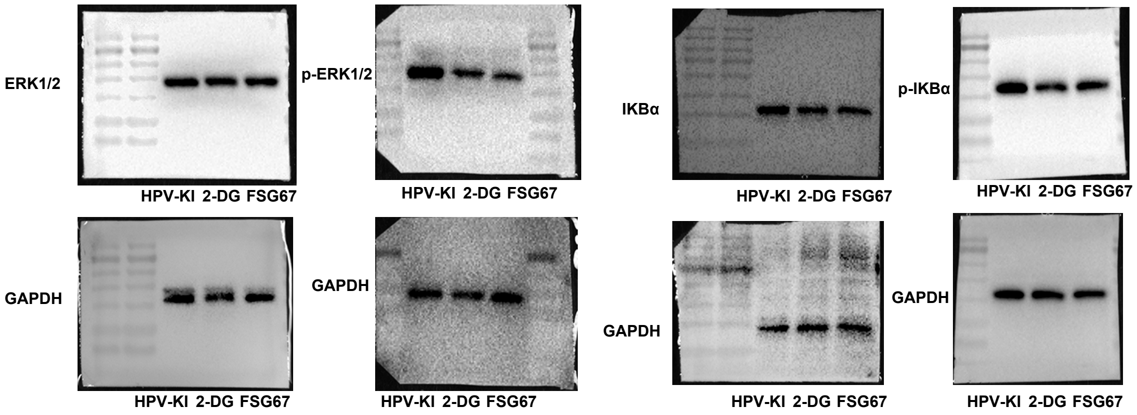

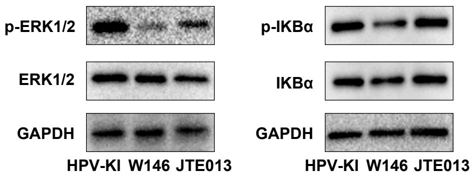

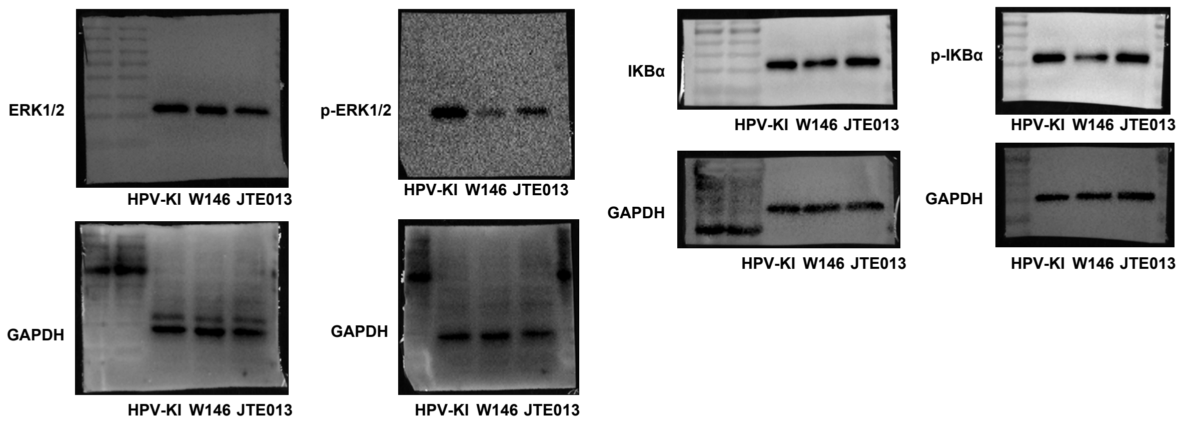

Supplement: Supplementary file 3 — Western blot unedited-revised [file 41419_2025_8195_MOESM3_ESM.pdf]
